# Phase and Amplitude Modes in the Anisotropic Dicke Model with Matter Interactions

**DOI:** 10.3390/e26070574

**Published:** 2024-07-03

**Authors:** Ricardo Herrera Romero, Miguel Angel Bastarrachea-Magnani

**Affiliations:** Departamento de Física, Universidad Autónoma Metropolitana-Iztapalapa, Av. Ferrocarril San Rafael Atlixco 186, Mexico City C.P. 09310, Mexico; ricardo.h.romero@outlook.com

**Keywords:** Dicke model, interacting qubits, quantum phase transitions, phase mode, amplitude mode, geometric phase

## Abstract

Phase and amplitude modes, also called polariton modes, are emergent phenomena that manifest across diverse physical systems, from condensed matter and particle physics to quantum optics. We study their behavior in an anisotropic Dicke model that includes collective matter interactions. We study the low-lying spectrum in the thermodynamic limit via the Holstein–Primakoff transformation and contrast the results with the semi-classical energy surface obtained via coherent states. We also explore the geometric phase for both boson and spin contours in the parameter space as a function of the phases in the system. We unveil novel phenomena due to the unique critical features provided by the interplay between the anisotropy and matter interactions. We expect our results to serve the observation of phase and amplitude modes in current quantum information platforms.

## 1. Introduction

Amplitude and phase modes are collective excitations of an order parameter usually studied in connection to a continuous symmetry breaking. For the U(1) symmetry, they are known as *massive* Anderson–Higgs (AH) [1,2] and *massless* Nambu–Goldstone (NG) modes [3,4], respectively, and are present in several systems, from condensed matter [5,6,7,8,9], quantum antiferromagnets [10,11,12] to particle physics [13,14,15]. Phase and amplitude modes help to describe critical phenomena such as quantum phase transitions (QPTs) [3], i.e., a parametric sudden change in the ground state’s properties of a quantum system [16,17]. The paradigmatic case is the quartic potential $V\left(\alpha \right)=r\alpha^{2}+g\alpha^{4}$ , expressed in terms of the scalar complex parameter α that possesses the U(1) continuous symmetry [16]. The parameter space is separated into two phases: the normal or non-ordered phase ( r,g>0 ), where the potential has the form of a single well with a minimum at α=0 , and the ordered phase ( r<0 ), where the potential has a Mexican hat form. The ground state is a degeneration of states with |α|>0 . In the ordered phase, fluctuations of the parameter quantify the two possible excitations: (1) the phase mode (NG), which occurs at a constant amplitude and has vanishing excitation energy, thus being massless; and (2) the amplitude mode (AH), which has a constant phase and possesses finite excitation energy, thus being massive. In general, both modes are hard to observe experimentally. The NG is particularly sensitive to symmetry deviations, as it gains finite excitation energy [18,19]. Likewise, the AH mode is challenging to observe because of its massive character. Ultracold atoms setups are particularly suitable for their observation and control due to their high tunability [19,20,21,22,23,24].

A standard example of a mean-field quantum phase transition (QPT) is the superradiant-to-normal transition of the Dicke Hamiltonian [25,26,27]. This model represents a collection of atoms, each simplified to a two-energy level transition, interacting with a single-mode radiation field within a cavity [28]. When the light–matter coupling strength exceeds a critical threshold, the QPT is indicated by a non-zero photon number expectation value in the thermodynamic limit. The Dicke Hamiltonian provides a comprehensive framework for describing spin–boson interactions by capturing the collective behavior of a set of two level systems or qubits in both equilibrium and non-equilibrium setups [29,30,31,32]. Its algebraic simplicity has allowed the investigation of fundamental topics such as quantum chaos [33,34,35,36], the quantum–classical correspondence [37,38,39,40], Excited-State Quantum Phase Transition (ESQPT) [41,42,43,44], non-equilibrium phenomena [30,45,46,47], and the physics of the ultrastrong light–matter coupling regime [48,49,50]. Thanks to this, the model has become a general framework to describe collective qubit effects in a vast array of systems, including ultracold atoms [51,52,53], superconducting qubits [54,55,56,57], nuclear physics [58], solid-state systems [59,60,61], and quantum dots [62]. Also, superradiance has been realized in superconducting qubits [63,64,65], cavity-assisted Raman transitions [66,67], bose-Einstein condensates in optical lattices [68,69,70,71,72], and unitary Fermi gases [73,74]. It is precisely in these setups where the amplitude and phase modes have been studied in connection to supersolid states exhibiting self-organization [22,75,76].

Under the rotating-wave approximation, the Dicke model becomes the Tavis–Cummings (TC) model [77], which is integrable in a classical sense, conserving the total number of excitations. This is reflected in the semi-classical limit, where a Mexican hat potential emerges in the superradiant phase [78]. In this symmetry-broken phase, many states cluster together to form a quasi-continuous band of energies, where the phase can take any value in [0,2π) . Hence, there is no cost of energy to displace the state or change the order parameter along the potential’s minima. Because of this, the TC model exhibits both a gapless phase mode and a gapped amplitude mode. Instead, the Dicke model has only a $Z_{2}$ symmetry. As a result, it has a doubly degenerate spectrum in the superradiant phase corresponding to a double-well semi-classical potential [78]. The pair of (symmetry-breaking) ground states correspond to those at the bottom of a potential, reflecting two possible values for the phase (0 and π ). Hence, the phase mode becomes gapped (Higgs-like) and corresponds to what has been called a roton-type mode [79,80,81] in analogy to the roton excitations in superfluid helium. The phase and amplitude modes in the Dicke model are called *polariton modes* [82], as well. A polariton is a hybrid quantum state emerging from strong light–matter interactions [83]. Because the phase and amplitude modes in the Dicke model represent the collective light–matter quantum state at low energy, they correspond to effective upper and lower polaritons [84,85]. The differences between the Dicke and TC models can be identified by studying the gap between the ground and first excited states. It has been studied for finite size [19,86], and in the thermodynamical limit [35,87]. Also, the phase and amplitude modes have been observed for large light–matter coupling [81,88]. A way to do this is by exploiting their singular behavior at the superradiant QPT [18]. The symmetry-breaking effect in the Dicke model has been measured experimentally [27,70], and employed for quantum sensing [89].

The so-called anisotropic, generalized, or unbalanced Dicke model [18,38,78,90,91,92] is a useful tool to explore the parametric change in the gap and understand the polariton modes as one passes from a continuous to a discrete symmetry, where the phase model can gain mass. There, one assumes a relative strength between the rotating (excitation conserving) and counter-rotating terms, allowing to tune the Hamiltonian between the TC and Dicke limits. Both the equilibrium [93,94,95,96] and non-equilibrium properties [72,97,98,99,100] of the anisotropic Dicke model have been studied, exhibiting a rich phase space and the presence of novel critical phenomena. Phase modes have been found in other extensions of the Dicke model, such as generalizations of the spin–boson coupling [101], Jahn–Teller–Dicke models [102], two-mode Dicke models [88,103,104,105], Jaynes–Cummings lattices [106], the three-level Dicke model [107,108], and also including matter–matter interactions [19].

In light of experimental progress in the combination of strong matter–matter interactions and ultrastrong light–matter systems, such as the recent realization of the Dicke model in a solid-state system with tunable spin–magnon interactions [109,110,111], in this work, we investigate the effects of collective matter interactions over the phase and amplitude or polariton modes in the anisotropic Dicke model. Collective matter interactions mediated by light introduce unique behavior in the Dicke model, such as first-order QPT [112,113,114,115,116,117,118], parametric shifts over the superradiant QPT [54,119,120], new quantum phases [117,121,122,123], and novel perspectives on quantum chaos [117,124], such as the amplification of the regularity-to-chaos transition [125]. Also, recently, the matter and light linear response has been investigated for this model [126,127] in the context of the interplay between cavity QED and magnetic systems.

To achieve our end, we employ the standard approach of applying the Holstein–Primakoff (HP) transformation to obtain a low-energy approximation of the excitation modes around the minima [79]. Given the limitations of the HP transformation [128], we interpret the results under the light of the semi-classical corresponding energy surfaces that can be obtained via coherent states [122]. Additionally, we calculate the geometric or Berry phase around the critical points and relate its behavior to the phase and amplitude modes [129,130]. The geometric phase possesses non-analytic behavior along a QPT, so it has been employed as a signature of criticality [131,132,133]. It has been observed experimentally in Heisenberg chains [134] and qubit systems [135,136]. It has been studied for the standard Dicke model [137,138,139] and extensions such as including two-photon processes [140], impurity coupling [141], and collective matter interactions in the *z* direction [142].

The article is organized as follows. In Section 2, we present the anisotropic Dicke Hamiltonian with matter interactions and details over its classical limit. Next, in Section 3, we obtain exact expressions for the low-lying polariton branches using the Holstein–Primakoff approximation. In Section 4, we discuss the gap behavior as a function of the different phases in the system. We calculate the geometrical phase for arbitrary circulations generated by the photon number or the relative population operator in Section 5. Finally, in Section 6, we present our conclusions and perspectives.

## 2. Anisotropic Dicke Hamiltonian with Matter Interactions

The anisotropic Dicke Hamiltonian including collective matter interactions is given by

(1) $${\hat{H}}_{\xi }=\omega {\hat{a}}^{†}\hat{a}+\omega_{0}{\hat{J}}_{z}+\frac{\gamma }{\sqrt{N}}\left[\left(\hat{a}{\hat{J}}_{+}+{\hat{a}}^{†}{\hat{J}}_{-}\right)+\xi \left({\hat{a}}^{†}{\hat{J}}_{+}+\hat{a}{\hat{J}}_{-}\right)\right]+\frac{1}{N}\left(\eta_{x}{\hat{J}}_{x}^{2}+\eta_{y}{\hat{J}}_{y}^{2}+\eta_{z}{\hat{J}}_{z}^{2}\right),$$

The first two terms denote the non-interacting parts of the Hamiltonian, where ω is the boson frequency, $\omega_{0}$ is the qubit energy splitting, ${\hat{a}}^{†}$ ( $\hat{a}$ ) is the creation (annihilation) boson operator, and ${\hat{J}}_{z}$ is the relative population of excited qubits, given that ${\hat{J}}_{z,x,y}$ are pseudospin operators representing the collective degrees of freedom of the set of *N* qubits, which follow the rules of the su(2)-algebra. The third term is the spin–boson interaction, with γ as the coupling strength and ξ as the anisotropy parameter, that allows tuning the relative strength of the counter-rotating term, so ξ=0 and ξ=1 are the TC and Dicke limits, respectively. Being a system made of the tensor product of two Hilbert spaces, the Hamiltonian follows both the algebraic properties of the su(2) and the Heisenberg–Weyl algebras. Like the standard Dicke model [78], it commutes with the parity operator $\hat{\Pi }=\exp\left(i\pi {\hat{n}}_{e}\right)$ , where $n_{e}={\hat{a}}^{†}\hat{a}+{\hat{J}}_{z}+j\hat{I}$ is the total number of the excitations operator. Because this is a discrete parity, the Hamiltonian is non-integrable in the classical sense, except for the TC limit, when the Hamiltonian commutes directly with ${\hat{n}}_{e}$ . Moreover, the Hamiltonian also commutes with the pseudospin length ${\hat{J}}^{2}=J_{x}^{2}+J_{y}^{2}+J_{z}^{2}$ , so the totally symmetric subspace, where j=N/2 , contains the ground-state of the collective system. The last term in Equation (1) accounts for the matter interactions, where $\eta_{i}$ with i=x,y,z are the couplings in each direction, whose meaning depends on the particular setup realizing the Dicke model, including Josephson dynamics [115,143,144], atomic dipolar couplings [119,145], optomechanical setups [146,147], or interactions between superconducting qubits [54,56,57,148,149]. A standard approach is regarding $\eta_{z}$ as the strength of collective on-site interaction and $\eta_{x}$ ( $\eta_{y}$ ) as a strength of the collective inter-qubit interactions. It is worth emphasizing that the relevant interacting parameters are $\Delta \eta_{z\mu }=\eta_{z}-\eta_{\mu }$ , with μ=x,y , given that the pseudospin length is conserved, and one can express one direction in terms of the others ${\hat{J}}_{z}^{2}=j\left(j+1\right)\hat{I}-{\hat{J}}_{x}^{2}-{\hat{J}}_{y}^{2}$ [122].

The Hamiltonian in Equation (1) commutes with the squared pseudospin length operator $\left[{\hat{H}}_{\xi },\hat{J^{2}}\right]$ , so the collective ground state lies in the totally symmetric subspace that corresponds to j=N/2 , and the Hilbert space of the system is effectively reduced to N+1 states. As a result, a classical corresponding Hamiltonian can be obtained by mapping the boson and collective pseudospin degrees of freedom to the classical limit via coherent states. To do so, one takes the value of the quantum Hamiltonian over the tensor product of Glauber |z〉 and Bloch |w〉 coherent states as trial states [38,40,78,150,151], where |0〉 and |j,−j〉 are the boson and pseudospin vacuum states, respectively [152]. In canonical classical variables for the boson (q,p) , $z=\sqrt{j/2}\left(q+ip\right)$ and the pseudospin $\left(j_{z},\phi \right)$ , $w=\sqrt{\left(1+j_{z}\right)/\left(1-j_{z}\right)}e^{-i\phi }$ , the classical corresponding Hamiltonian reads [122]

(2) $$\begin{array}{c} H_{\xi }^{\left(cl\right)}=\frac{\omega }{2}\left(q^{2}+p^{2}\right)+j_{z}\left(\omega_{0}+\frac{\eta_{z}j_{z}}{2}\right)+\frac{1}{2}\left(1-j_{z}^{2}\right)\left(\eta_{x}\cos^{2}\phi +\eta_{y}\sin^{2}\phi \right)\\ +\gamma \sqrt{1-j_{z}^{2}}\left[(1+\xi )q\cos\phi -(1-\xi )p\sin\phi \right]. \end{array}$$

A standard method to determine quantum phases in this class of systems with collective degrees of freedom is to analyze the extreme points of the corresponding classical energy surfaces under catastrophe theory [153,154], here, the one in Equation (2). Because the ground state is well-described by coherent states as trial states, dramatic changes in the energy surface’s minima point out the existence of ground-state QPT. This method is extended to other extreme points on the energy surface to identify excited-state QPT or ESQPT [78,93,117]. In the following, we will focus only on the absolute minima of the energy surfaces, as we are interested in polariton modes. We employ the classical energy surfaces to interpret the behavior of these modes across the parameter space. We picture the surfaces in a set of variables $\left(u,v\right)=\arccos\left(-j_{z}\right)\left(\cos\phi ,\sin\phi \right)$ that, by using Hamilton equations to eliminate the bosonic quadratures *q* and *p*, allows one to visualize the fixed points in the collective pseudospin space alone [78,122]. In this picture, the energy surface reads as

(3) $$\begin{array}{c} E\left(\xi ,u,v\right)=\omega_{0}\sin^{2}\sqrt{u^{2}+v^{2}}\frac{1}{2\left(u^{2}+v^{2}\right)}\left[u^{2}\left(\frac{\eta_{x}}{\omega_{0}}-f_{\xi +}\right)+v^{2}\left(\frac{\eta_{y}}{\omega_{0}}-f_{\xi -}\right)\right]-\\ \omega_{0}\cos\sqrt{u^{2}+v^{2}}\left(1-\frac{\eta_{z}}{2\omega_{0}}\cos\sqrt{u^{2}+v^{2}}\right). \end{array}$$

where $f_{\xi \pm }=\gamma^{2}/\gamma_{\xi \pm }^{c}$ and $\gamma_{\xi \pm }^{c}=\sqrt{\omega \omega_{0}}/\left(1\pm \xi \right)$ are the critical couplings for the superradiant- (±) phases as detailed below.

The anisotropy introduces new quantum phases to the standard Dicke model that matter–matter interactions then modify. For zero matter interactions ( $\eta_{i}=0$ ), the Dicke ( ξ=1 ) and TC ( ξ=0 ) models exhibit only two phases, the normal (non-ordered) and the superradiant (ordered), separated by the critical coupling $\gamma_{\xi +}^{c}$ . The anisotropy modifies the energy landscape such that two different superimposed phases appear, the superradiant- (+) and superradiant- (−) phases, the latter with a critical coupling $\gamma_{\xi -}^{c}$ [44,93,94]. These phases are also called the electric and magnetic superradiance, with a different coupling combination for the counter and counter-rotating terms [18,92]. Each phase has its extreme points, but only one predominates as the ground state, depending on which of the two terms, the rotating or the counter-rotating, is stronger. For our case of study ξ∈[0,1] , the superradiant-*x* phase defines the ground state.

In the presence of interactions ( $\eta_{i}\neq 0$ ) but in the absence of light–matter coupling ( γ=0 ), one obtains a Lipkin–Meshkov–Glick (LMG) Hamiltonian [155,156,157]. Hence, the anisotropic Dicke Hamiltonian inherits the algebraic properties and critical phenomena from the LMG [158,159,160,161,162,163,164,165,166]. There are three major modifications to the ground-state quantum phase diagram of the anisotropic Dicke model due to matter interactions [122]. First, interactions in the *x* and *y* direction produce a deformation, creating a *deformed* normal phase [92]. Second, the superradiant- (+) (superradiant- (−) ) phase transforms into the superradiant-*x* (superradiant-*y*), and the critical coupling is modified to $\gamma_{\xi x}^{c}={\left(1+\Delta \eta_{zx}/\omega_{0}\right)}^{1/2}\gamma_{\xi +}^{c}$ ( $\gamma_{\xi y}^{c}={\left(1+\Delta \eta_{zy}/\omega_{0}\right)}^{1/2}\gamma_{\xi -}^{c}$ ) [122]. Finally, while $\eta_{z}$ produces an overall energy shift, in the case of the Dicke limit ( ξ=1 ), a new phase emerges, the deformed phase, which suppresses superradiance for $\Delta \eta_{zy}\geq 1$ and is the source of a first-order QPT for arbitrary coupling [117]. In the next section, we discuss the behavior of the polariton modes in each of these quantum phases.

## 3. Phase and Amplitude Modes in the Anisotropic Dicke Model

The Holstein–Primakoff transformation (HPT) [167] provides a quadratic approximation to the lower energy modes of Equation (1) in the thermodynamic limit ( N→∞ ). After diagonalizing the quadratic, approximated Hamiltonian, the resulting branches correspond to the polariton modes or mean-field low-lying excitations of the photon–matter quantum superposition [18,80]. The low-lying spectrum can also be obtained by linearization of the classical equations of motion, i.e., calculating the small oscillations around the minima [168,169]. The HPT reads

(4) $$\begin{array}{c} {\hat{J}}_{z}={\hat{b}}^{†}\hat{b}-j,\;\;\;{\hat{J}}_{+}=\sqrt{2j}\;{\hat{b}}^{†}\sqrt{1-\frac{{\hat{b}}^{†}\hat{b}}{2j}},\;\;\;{\hat{J}}_{-}=\sqrt{2j}\;\sqrt{1-\frac{{\hat{b}}^{†}\hat{b}}{2j}}\;\hat{b}. \end{array}$$

where $\hat{b}$ ( ${\hat{b}}^{†}$ ) is an annhilation (creation) boson operator such that $[\hat{b},{\hat{b}}^{†}]=I$ . First, by substituting Equation (4) in Equation (1), one obtains

(5) $$\begin{array}{c} \hat{H}=\omega {\hat{a}}^{†}\hat{a}+\omega_{0}\left({\hat{b}}^{†}\hat{b}-j\right)\\ +\gamma \left[\left(\hat{a}{\hat{b}}^{†}\sqrt{1-\frac{{\hat{b}}^{†}\hat{b}}{2j}}+{\hat{a}}^{†}\sqrt{1-\frac{{\hat{b}}^{†}\hat{b}}{2j}}\hat{b}\right)+\xi \left({\hat{a}}^{†}{\hat{b}}^{†}\sqrt{1-\frac{{\hat{b}}^{†}\hat{b}}{2j}}+\hat{a}\sqrt{1-\frac{{\hat{b}}^{†}\hat{b}}{2j}}\hat{b}\right)\right]\\ \frac{1}{4}\left[\eta_{x}{\left({\hat{b}}^{†}\sqrt{1-\frac{{\hat{b}}^{†}\hat{b}}{2j}}+\sqrt{1-\frac{{\hat{b}}^{†}\hat{b}}{2j}}\hat{b}\right)}^{2}-\eta_{y}{\left({\hat{b}}^{†}\sqrt{1-\frac{{\hat{b}}^{†}\hat{b}}{2j}}-\sqrt{1-\frac{{\hat{b}}^{†}\hat{b}}{2j}}\hat{b}\right)}^{2}\right]+\frac{\eta_{z}}{2j}{\left({\hat{b}}^{†}\hat{b}-j\right)}^{2} \end{array}$$

Next, to study the excitation modes, we employ a mean field approximation by displacing both $\hat{a}$ and $\hat{b}$ bosonic operators

(6) $$\begin{array}{c} {\hat{a}}^{†}\to {\hat{c}}^{†}+\alpha \sqrt{2j},\;\;\;\mathrm{and}\;\;\;{\hat{b}}^{†}\to {\hat{d}}^{†}-\beta \sqrt{2j}. \end{array}$$

where α,β∈C are scaled displacements. The square roots in the HPT become

(7) $$\begin{array}{c} \sqrt{1-\frac{{\hat{b}}^{†}\hat{b}}{2j}}=\sqrt{1-\frac{{\hat{d}}^{†}\hat{d}-\beta \sqrt{2j}\left({\hat{d}}^{†}+\hat{d}\right)+2j\beta^{2}}{2j}}=\sqrt{k}\sqrt{\xi_{0}},\\ k=1-\beta^{2},\;\;\;\sqrt{\xi_{0}}=\sqrt{1-\frac{{\hat{d}}^{†}\hat{d}-\beta \sqrt{2j}\left({\hat{d}}^{†}+\hat{d}\right)+2j\beta^{2}}{2jk}} \end{array}$$

Next, we make use of the thermodynamic limit N→∞ . Here, the customary assumption is that $\langle {\hat{b}}^{†}\hat{b}\rangle \ll 2j$ , so $\sqrt{1-{\hat{b}}^{†}\hat{b}/2j}≃1-{\hat{b}}^{†}\hat{b}$ . In particular,

(8) $$\begin{array}{c} \sqrt{\xi_{0}}≃1-\frac{{\hat{d}}^{†}\hat{d}-\beta \sqrt{2j}\left({\hat{d}}^{†}\hat{d}\right)}{4jk}-\frac{\beta^{2}{\left({\hat{d}}^{†}+\hat{d}\right)}^{2}}{16jk^{2}} \end{array}$$

By keeping only terms up to the quadratic order, the Hamiltonian becomes

(9) $$\begin{array}{c} \hat{H}=\omega {\hat{c}}^{†}\hat{c}+\left[\omega_{0}+\gamma \frac{\alpha \beta }{\sqrt{k}}\left(1+\xi \right)+\eta_{z}\left(2\beta^{2}-1\right)-\beta^{2}\eta_{x}\right]{\hat{d}}^{†}\hat{d}\\ +\left[\omega \alpha -\gamma \sqrt{k}\beta \left(1+\xi \right)\right]\sqrt{2j}\left({\hat{c}}^{†}+\hat{c}\right)\\ +\left[-\omega_{0}\beta +\gamma \alpha \left(\frac{k-\beta^{2}}{\sqrt{k}}\right)\left(1+\xi \right)-\eta_{x}k\beta \left(1-\frac{\beta^{2}}{k}\right)-\eta_{z}\left(2\beta^{2}-1\right)\beta \right]\sqrt{2j}\left({\hat{d}}^{†}+\hat{d}\right)\\ +\left[\gamma \frac{\alpha \beta }{4\sqrt{k}k}\left(2-\beta^{2}\right)\left(1+\xi \right)+\eta_{x}\frac{k}{4}\left(1-\frac{4\beta^{2}}{k}\right)+\eta_{z}\beta^{2}\right]{\left({\hat{d}}^{†}+\hat{d}\right)}^{2}-\eta_{y}\frac{k}{4}{\left({\hat{d}}^{†}-\hat{d}\right)}^{2}\\ -\gamma \left[\frac{\beta^{2}}{2\sqrt{k}}\left(1+\xi \right)\right]\left({\hat{c}}^{†}+\hat{c}\right)\left({\hat{d}}^{†}+\hat{d}\right)+\gamma \sqrt{\xi_{0}}\left[\left(\hat{c}d^{†}+{\hat{c}}^{†}\hat{d}\right)+\xi \left({\hat{c}}^{†}{\hat{d}}^{†}+\hat{c}\hat{d}\right)\right]\\ +\left[\omega \alpha^{2}+\omega_{0}\beta^{2}-\frac{\omega_{0}}{2}-\gamma \sqrt{k}2\alpha \beta \left(1+\xi \right)+\eta_{x}k\beta^{2}+\eta_{z}{\left(\beta^{2}-\frac{1}{2}\right)}^{2}\right]2j\\ -\gamma \frac{\alpha \beta }{2\sqrt{k}}\left(1+\xi \right)+\eta_{x}\frac{\beta^{2}}{2}, \end{array}$$

### 3.1. Deformed Normal Phases

The deformed normal phases are characterized by a zero expectation value of photon and matter excitations. Thus, by setting α,β→0 , we obtain

(10) $$\begin{array}{c} \hat{H}=\omega {\hat{c}}^{†}\hat{c}+\omega_{0}\left({\hat{d}}^{†}\hat{d}-j\right)+\gamma \left[\left(\hat{c}{\hat{d}}^{†}+{\hat{c}}^{†}\hat{d}\right)+\xi \left({\hat{c}}^{†}{\hat{d}}^{†}+\hat{c}\hat{d}\right)\right]+\\ \frac{1}{4}\left[\eta_{x}{\left({\hat{d}}^{†}+\hat{d}\right)}^{2}-\eta_{y}{\left({\hat{d}}^{†}-\hat{d}\right)}^{2}\right]+\frac{\eta_{z}}{2j}{\left({\hat{d}}^{†}\hat{d}-j\right)}^{2}. \end{array}$$

Next, one writes the Hamiltonian in terms of the boson quadratures [79]

(11) $$\begin{array}{c} \hat{c}=\sqrt{\frac{\omega }{2}}\left(\hat{x}+\frac{i}{\omega }{\hat{p}}_{x}\right),\;\;\;\hat{d}=\sqrt{\frac{\omega_{0}}{2}}\left(\hat{y}+\frac{i}{\omega_{0}}{\hat{p}}_{y}\right), \end{array}$$

By substituting Equation (11), the Hamiltonian is now represented as follows:

(12) $$\begin{array}{c} \hat{H}=\frac{1}{2}\left[\omega^{2}{\hat{x}}^{2}+\omega_{zx}^{2}{\hat{y}}^{2}+{\hat{p}}_{x}^{2}+\omega_{zy}^{2}{\hat{p}}_{y}^{2}+\frac{\gamma }{\gamma_{\xi +}}2\omega \omega_{0}\hat{x}\hat{y}+\frac{\gamma }{\gamma_{\xi -}}2{\hat{p}}_{x}{\hat{p}}_{y}-\epsilon_{0}\right]. \end{array}$$

where

(13) $$\begin{array}{c} \epsilon_{0}=\omega +\omega_{0}\left(1-\frac{\eta_{z}}{\omega_{0}}\right)+2j\omega_{0}\left(1-\frac{\eta_{z}}{2\omega_{0}}\right), \end{array}$$

We consider the following dimensionless frequencies containing the matter interaction strengths

(14) $$\begin{array}{c} {\tilde{\omega }}_{zx}=\sqrt{1-\frac{\Delta \eta_{zx}}{\omega_{0}}},\;\;\;\mathrm{and}\;\;\;{\tilde{\omega }}_{zy}=\sqrt{1-\frac{\Delta \eta_{zy}}{\omega_{0}}}. \end{array}$$

Next, two rotations are performed to eliminate the cross terms $\hat{x}\hat{y}$ and ${\hat{p}}_{x}{\hat{p}}_{y}$ . We will apply a rotation with angle $\theta_{1}$ for the variables $\hat{x}$ and $\hat{y}$ , and another with angle $\theta_{2}$ for ${\hat{p}}_{x}$ and ${\hat{p}}_{y}$ :

(15) $$\begin{array}{c} \left(\begin{array}{c} \hat{x}\\ \hat{y} \end{array}\right)\left(\begin{array}{cc} \cos\theta_{1} & \sin\theta_{1}\\ -\sin\theta_{1} & \cos\theta_{1} \end{array}\right)\left(\begin{array}{c} {\hat{q}}_{1}\\ {\hat{q}}_{2} \end{array}\right),\;\;\;\left(\begin{array}{c} {\hat{p}}_{x}\\ {\hat{p}}_{y} \end{array}\right)\left(\begin{array}{cc} \cos\theta_{2} & \sin\theta_{2}\\ -\sin\theta_{2} & \cos\theta_{2} \end{array}\right)\left(\begin{array}{c} {\hat{p}}_{1}\\ {\hat{p}}_{2} \end{array}\right). \end{array}$$

The following conditions arise:

(16) $$\begin{array}{c} \tan2\theta_{1}=2f_{\xi x}^{1/2}\frac{\omega \omega_{0}{\tilde{\omega }}_{zx}}{\omega_{0}^{2}{\tilde{\omega }}_{zx}^{2}-\omega^{2}},\;\;\;\mathrm{and}\;\;\;\tan2\theta_{2}=2f_{\xi x}^{1/2}\left(\frac{1-\xi }{1+\xi }\right)\frac{{\tilde{\omega }}_{zx}}{{\tilde{\omega }}_{zy}^{4}-1}, \end{array}$$

where $f_{\xi x}={\left(\gamma /\gamma_{\xi x}\right)}^{2}$ , and the critical coupling indicating the onset of superradiance is

(17) $$\begin{array}{c} \gamma_{\xi x}^{c}=\gamma_{\xi +}^{c}{\tilde{\omega }}_{zx}. \end{array}$$

Substituting these conditions yields a two-mode decoupled Hamiltonian, determining the energies $\epsilon_{1\pm }^{N}$ that represent the low-lying excitation modes:

(18) $$\begin{array}{c} \hat{H}=\frac{1}{2}\left[{\left(\epsilon_{1-}^{N}\right)}^{2}{\hat{q}}_{1}^{2}+{\left(\epsilon_{1+}^{N}\right)}^{2}{\hat{q}}_{2}^{2}+{\left(\epsilon_{2-}^{N}\right)}^{2}{\hat{p}}_{1}^{2}+{\left(\epsilon_{2+}^{N}\right)}^{2}{\hat{p}}_{2}^{2}-\epsilon_{0}\right], \end{array}$$

with energies given by

(19) $$\begin{array}{c} \epsilon_{1\pm }^{N}=\sqrt{\frac{1}{2}\left[\left(\omega^{2}+\omega_{0}^{2}{\tilde{\omega }}_{zx}^{2}\right)\pm \sqrt{{\left(\omega^{2}-\omega_{0}^{2}{\tilde{\omega }}_{zx}^{2}\right)}^{2}+4\omega^{2}\omega_{0}^{2}{\tilde{\omega }}_{zx}^{2}f_{\xi x}}\right]}, \end{array}$$

(20) $$\begin{array}{c} \epsilon_{2\pm }^{N}=\sqrt{\frac{1}{2}\left[\left(1+{\tilde{\omega }}_{zy}^{2}\right)\pm \sqrt{{\left(1-{\tilde{\omega }}_{zy}^{2}\right)}^{2}+4{\tilde{\omega }}_{zy}^{2}g_{\xi y}}\right]} \end{array}$$

where $g_{\xi y}={\left(\gamma /\gamma_{\xi y}\right)}^{2}$ , with

(21) $$\begin{array}{c} \gamma_{\xi y}=\gamma_{\xi -}^{c}{\tilde{\omega }}_{zy} \end{array}$$

the critical coupling of the superradiant-*y* behavior. It is convenient to rewrite Equation (18) in a second quantized form in order to obtain a decoupled Hamiltonian with new bosonic excitations ${\hat{a}}_{i}$ ( ${\hat{a}}_{i}^{†}$ ) with i=1,2 following $[{\hat{a}}_{i},{\hat{a}}_{i}^{†}]=I$ . They read

(22) $$\begin{array}{c} {\hat{q}}_{1}=\frac{\left({\hat{a}}_{1}^{†}+{\hat{a}}_{1}\right)}{\sqrt{2\omega_{N_{-}}}},\;\;\;{\hat{p}}_{1}=i\sqrt{\frac{\omega_{N_{-}}}{2}}\left({\hat{a}}_{1}^{†}-{\hat{a}}_{1}\right), \end{array}$$

(23) $$\begin{array}{c} {\hat{q}}_{2}=\frac{\left({\hat{a}}_{2}^{†}+{\hat{a}}_{2}\right)}{\sqrt{2\omega_{N_{+}}}},\;\;\;{\hat{p}}_{2}=i\sqrt{\frac{\omega_{N_{+}}}{2}}\left({\hat{a}}_{2}^{†}-{\hat{a}}_{2}\right), \end{array}$$

where $\omega_{N_{-}}=\epsilon_{1-}^{N}/\epsilon_{2-}^{N}$ , and $\omega_{N_{+}}=\epsilon_{1+}^{N}/\epsilon_{2+}^{N}$ . Thus, we obtain the quantum oscillation modes and the low-energy spectrum. It is essential to highlight that these expressions depend on the $\Delta \eta_{xz}$ and $\Delta \eta_{yz}$ parameters, allowing us to modulate the behavior of the modes both in the normal phase and in the superradiant phase as we will see later on. Finally, the Hamiltonian reads

(24) $$\begin{array}{c} \hat{H}=\epsilon_{0}^{N}+\epsilon_{-}^{N}{\hat{a}}_{1}^{†}{\hat{a}}_{1}+\epsilon_{+}^{N}{\hat{a}}_{2}^{†}{\hat{a}}_{2}, \end{array}$$

with

(25) $$\begin{array}{c} \epsilon_{0}^{N}=\frac{1}{2}\left(\epsilon_{-}^{N}+\epsilon_{+}^{N}-\epsilon_{0}\right) \end{array}$$

The phase $\epsilon_{-}^{N}$ and amplitude $\epsilon_{+}^{N}$ modes in the deformed normal phases become

(26) $$\begin{array}{c} \epsilon_{-}^{N}=\epsilon_{2-}^{N}\epsilon_{1-}^{N},\;\;\;\epsilon_{+}^{N}=\epsilon_{2+}^{N}\epsilon_{1+}^{N}. \end{array}$$

### 3.2. Superradiant Phases

Now, we consider the case where α,β≠0 . To obtain a quadratic Hamiltonian, we need to eliminate the linear terms in $\hat{c},{\hat{c}}^{†}$ , and $\hat{d},{\hat{d}}^{†}$ from Equation (3). Hence, we obtain the following conditions determining the values of α and β ,

(27) $$\begin{array}{c} \omega \alpha -\gamma \sqrt{k}\beta \left(1+\xi \right)=0, \end{array}$$

(28) $$\begin{array}{c} -\omega_{0}\beta +\gamma \alpha \left(\frac{k-\beta^{2}}{\sqrt{k}}\right)\left(1+\xi \right)-\eta_{x}k\beta \left(1-\frac{\beta^{2}}{k}\right)-\eta_{z}\left(2\beta^{2}-1\right)\beta =0 \end{array}$$

Solving the system of equations leads to

(29) $$\begin{array}{c} \alpha =\frac{\gamma (1+\xi )}{2\omega }\sqrt{1-\mu_{x}^{2}}\;\;\;\mathrm{and}\;\;\;\beta =\sqrt{\frac{1}{2}\left(1-\mu_{x}\right)}, \end{array}$$

where

(30) $$\begin{array}{c} \mu_{x}={\left[{\tilde{\omega }}_{zx}^{2}\left(f_{\xi x}-1\right)+1\right]}^{-1}. \end{array}$$

The critical coupling $\gamma_{\xi x}^{c}$ in Equation (17) for the existence of superradiant behavior is determined by $\mu_{x}=1$ because for $\mu_{x}>1$ , α and β in Equation (29) become complex. Writing the resulting Hamiltonian in terms of Equation (29), we have:

(31) $$\begin{array}{c} \hat{H}=\omega {\hat{c}}^{†}\hat{c}+\omega_{A}{\hat{d}}^{†}\hat{d}+\omega_{B}{\left({\hat{d}}^{†}+\hat{d}\right)}^{2}+\omega_{C}{\left({\hat{d}}^{†}-\hat{d}\right)}^{2}+\omega_{D}\left({\hat{c}}^{†}+\hat{c}\right)\left({\hat{d}}^{†}+\hat{d}\right)\\ +\omega_{E}\left[\left(cd^{†}+{\hat{c}}^{†}\hat{d}\right)+\xi \left({\hat{c}}^{†}{\hat{d}}^{†}+\hat{c}\hat{d}\right)\right]+\omega_{F} \end{array}$$

where we have defined a set of reduced variables

$$\begin{array}{c} \omega_{A}=\frac{\omega_{0}}{2}\left(\frac{1}{\mu_{x}}-\frac{\eta_{z}}{\omega_{0}}\right)\left(1+\mu_{x}\right),\\ \omega_{B}=\frac{\omega_{0}}{2}\frac{1-\mu_{x}}{4}\left[\frac{1}{1+\mu_{x}}\frac{3+\mu_{x}}{\mu_{x}}+\frac{\eta_{z}}{\omega_{0}}\frac{1+3\mu_{x}}{1+\mu_{x}}+\frac{\eta_{x}}{\omega_{0}}\frac{4\mu_{x}^{2}}{1-\mu_{x}^{2}}\right],\\ \omega_{C}=-\frac{\omega_{0}}{8}\frac{\eta_{y}}{\omega_{0}}\left(1+\mu_{x}\right),\;\;\;\omega_{D}=-\frac{\sqrt{2}}{4}f_{\xi x}^{1/2}\sqrt{\omega \omega_{0}}{\tilde{\omega }}_{zx}\frac{1-\mu_{x}}{\sqrt{1+\mu_{x}}},\;\;\;\omega_{E}=\gamma \sqrt{\frac{1}{2}\left(1+\mu_{x}\right)},\\ \omega_{F}=-\frac{\omega_{0}}{2}\left\{\left[\frac{1-\mu_{x}^{2}}{2\mu_{x}}+\left(\mu_{x}-\frac{\eta_{z}}{2\omega_{0}}\right)\right]2j+\frac{1}{2}\left[\frac{1}{\mu_{x}}-\frac{\eta_{z}}{\omega_{0}}\right]\left(1-\mu_{x}\right)\right\}. \end{array}$$

Just as we did in the deformed normal phases, we will proceed to diagonalize the Hamiltonian in Equation (31) by transforming it into quadratures and then applying rotations that decoupled them. This transformation reads

(32) $$\begin{array}{c} \hat{c}=\sqrt{\frac{\omega }{2}}\left(\hat{x}+\frac{i}{\omega }{\hat{p}}_{x}\right),\;\;\;\hat{d}=\sqrt{\frac{\omega_{A}}{2}}\left(\hat{y}+\frac{i}{\omega_{A}}{\hat{p}}_{y}\right),\;\;\; \end{array}$$

so the Hamiltonian is expressed as

(33) $$\begin{array}{c} \hat{H}=\frac{1}{2}\left(\omega^{2}{\hat{x}}^{2}+\omega_{A}^{2}\chi_{+}{\hat{y}}^{2}+{\hat{p}}_{x}^{2}+\chi_{-}{\hat{p}}_{y}^{2}+\sqrt{\omega \omega_{A}}\kappa_{+}\hat{x}\hat{y}+\frac{\kappa_{-}}{\sqrt{\omega \omega_{A}}}{\hat{p}}_{x}{\hat{p}}_{y}-\epsilon_{1}\right). \end{array}$$

where

(34) $$\begin{array}{c} \epsilon_{1}=\omega_{0}\left[\frac{1-\mu_{x}^{2}}{2\mu_{x}}+\left(\mu_{x}-\frac{\eta_{z}}{2\omega_{0}}\right)\right]2j+\omega_{0}\left(\frac{1}{\mu_{x}}-\frac{\eta_{z}}{\omega_{0}}\right)+\omega \end{array}$$

Next, we consider the following variable changes

$$\begin{array}{c} \chi_{+}=\left(1+4\frac{\omega_{B}}{\omega_{A}}\right),\;\;\;\kappa_{+}=4\omega_{D}+\sqrt{2}f_{\xi x}^{1/2}\sqrt{\omega \omega_{0}}{\tilde{\omega }}_{zx}\sqrt{1+\mu_{x}},\\ \chi_{-}=\left(1-4\frac{\omega_{C}}{\omega_{A}}\right),\;\;\;\kappa_{-}=\sqrt{2}g_{\xi y}^{1/2}\sqrt{\omega \omega_{0}}{\tilde{\omega }}_{zy}\sqrt{1+\mu_{x}}. \end{array}$$

Again, we apply two rotations, $\phi_{1}$ for *x* and *y*, and $\phi_{2}$ for $p_{x}$ and $p_{y}$ ,

(35) $$\begin{array}{c} \left(\begin{array}{c} \hat{x}\\ \hat{y} \end{array}\right)\left(\begin{array}{cc} \cos\phi_{1} & \sin\phi_{1}\\ -\sin\phi_{1} & \cos\phi_{1} \end{array}\right)\left(\begin{array}{c} {\hat{q}}_{1}\\ {\hat{q}}_{2} \end{array}\right),\;\;\;\left(\begin{array}{c} {\hat{p}}_{x}\\ {\hat{p}}_{y} \end{array}\right)\left(\begin{array}{cc} \cos\phi_{2} & \sin\phi_{2}\\ -\sin\phi_{2} & \cos\phi_{2} \end{array}\right)\left(\begin{array}{c} {\hat{p}}_{1}\\ {\hat{p}}_{2} \end{array}\right). \end{array}$$

We obtain the following conditions for the angles $\phi_{1}$ and $\phi_{2}$ from eliminating the crossed terms ${\hat{q}}_{1}{\hat{q}}_{2}$ and ${\hat{p}}_{1}{\hat{p}}_{2}$ ,

(36) $$\begin{array}{c} \tan2\phi_{1}=\frac{\sqrt{\omega \omega_{A}}\kappa_{+}}{\omega_{A}^{2}\chi_{+}-\omega^{2}}\;\;\;y\;\;\;\tan2\phi_{2}=\frac{\kappa_{-}}{\sqrt{\omega \omega_{A}}\left(\chi_{-}-1\right)}. \end{array}$$

The Hamiltonian reduces to:

(37) $$\begin{array}{c} \hat{H}=\frac{1}{2}\left[{\left(\epsilon_{1-}^{S}{\hat{q}}_{1}\right)}^{2}+{\left(\epsilon_{1+}^{S}{\hat{q}}_{2}\right)}^{2}+{\left(\epsilon_{2-}^{S}{\hat{p}}_{1}\right)}^{2}+{\left(\epsilon_{2+}^{S}{\hat{p}}_{2}\right)}^{2}-\epsilon_{1}\right], \end{array}$$

with energies given by

(38) $$\begin{array}{c} \epsilon_{1\pm }^{S}=\sqrt{\frac{1}{2}\left(\left(\omega^{2}+\omega_{A}^{2}\chi_{+}\right)\pm \sqrt{{\left(\omega_{A}^{2}\chi_{+}-\omega^{2}\right)}^{2}+\omega \omega_{A}\kappa_{+}^{2}}\right)}, \end{array}$$

(39) $$\begin{array}{c} \epsilon_{2\pm }^{S}=\sqrt{\frac{1}{2}\left(\left(1+\chi_{-}\right)\pm \sqrt{{\left(\chi_{-}-1\right)}^{2}+\frac{\kappa_{-}^{2}}{\omega \omega_{A}}}\right)}. \end{array}$$

We decouple the Hamiltonian using quadratures:

$$\begin{array}{c} {\hat{q}}_{1}=\frac{\left({\hat{a}}_{1}^{†}+{\hat{a}}_{1}\right)}{\sqrt{2\omega_{S_{-}}}},\;\;\;{\hat{p}}_{1}=i\sqrt{\frac{\omega_{S_{-}}}{2}}\left({\hat{a}}_{1}^{†}-{\hat{a}}_{1}\right),\\ {\hat{q}}_{2}=\frac{\left({\hat{a}}_{2}^{†}+{\hat{a}}_{2}\right)}{\sqrt{2\omega_{S_{+}}}},\;\;\;{\hat{p}}_{2}=i\sqrt{\frac{\omega_{S_{+}}}{2}}\left({\hat{a}}_{2}^{†}-{\hat{a}}_{2}\right), \end{array}$$

where, as in the deformed normal case, $\omega_{S_{-}}=\epsilon_{1-}^{S}/\epsilon_{2-}^{S}$ , $\omega_{S_{+}}=\epsilon_{1+}^{S}/\epsilon_{2+}^{S}$ . Finally, the Hamiltonian reads in second quantized form as

(40) $$\begin{array}{c} \hat{H}=\epsilon_{0}^{S}+\epsilon_{-}^{S}{\hat{a}}_{1}^{†}{\hat{a}}_{1}+\epsilon_{+}^{S}{\hat{a}}_{2}^{†}{\hat{a}}_{2} \end{array}$$

with

(41) $$\begin{array}{c} \epsilon_{0}^{S}=\frac{1}{2}\left(\epsilon_{-}^{S}+\epsilon_{+}^{S}-\epsilon_{1}\right), \end{array}$$

with the phase $\epsilon_{-}^{S}$ and amplitude $\epsilon_{+}^{S}$ modes being

(42) $$\begin{array}{c} \epsilon_{-}^{S}=\epsilon_{2-}^{S}\epsilon_{1-}^{S},\;\;\;\epsilon_{+}^{S}=\epsilon_{2+}^{S}\epsilon_{1+}^{S}. \end{array}$$

### 3.3. Deformed Phase

Here, we comment on the deformed phase in the Dicke limit ( ξ=1 ) that suppresses the superradiant-*y* phase [117,122]. It exists independently of the spin–boson coupling γ , while $\Delta \eta_{zy}\leq \omega_{0}$ , and it is characterized by the zero expectation value of the photon number but is not a normal phase. This can be recognized in the energy surface by a π rotation of the extreme points. If we take ξ=1 in Equations (19) and (38), $g_{\xi y}\to 0$ , we obtain

(43) $$\begin{array}{c} \epsilon_{2\pm }^{N}=\sqrt{1-\left(1\mp 1\right)\frac{\Delta \eta_{zy}}{2\omega_{0}}},\;\;\;\;\epsilon_{2\pm }^{S}=\sqrt{\frac{\left(1\mp 1\right)\left(1-\frac{\Delta \eta_{zy}\mu_{x}}{\omega_{0}}\right)+\left(1\pm 1\right)\left(1-\frac{\eta_{z}\mu_{x}}{\omega_{0}}\right)}{2\left(1-\frac{\eta_{z}\mu_{x}}{\omega_{0}}\right)}}. \end{array}$$

Because $\epsilon_{\pm }^{S,N}=\epsilon_{2\pm }^{S,N}\epsilon_{1\pm }^{S,N}$ , the phase mode becomes undefined when $\Delta \eta_{zy}\geq \omega_{0}$ , or $\Delta \eta_{zy}\mu_{x}\geq \omega_{0}$ in the normal and superradiant phases, respectively, and the amplitude mode turns to $\epsilon_{-}^{N,S}=\epsilon_{1-}^{N,S}$ .

## 4. Role of Matter–Matter Interactions

Next, we explore the behavior of the phase and amplitude modes as a function of the matter–matter interaction parameters for the Dicke ( ξ=1 ), TC ( ξ=0 ) and an anisotropic case, particularly ξ=0.5 as an example.

### 4.1. Absence of Matter–Matter Interactions

**Figure 1:**
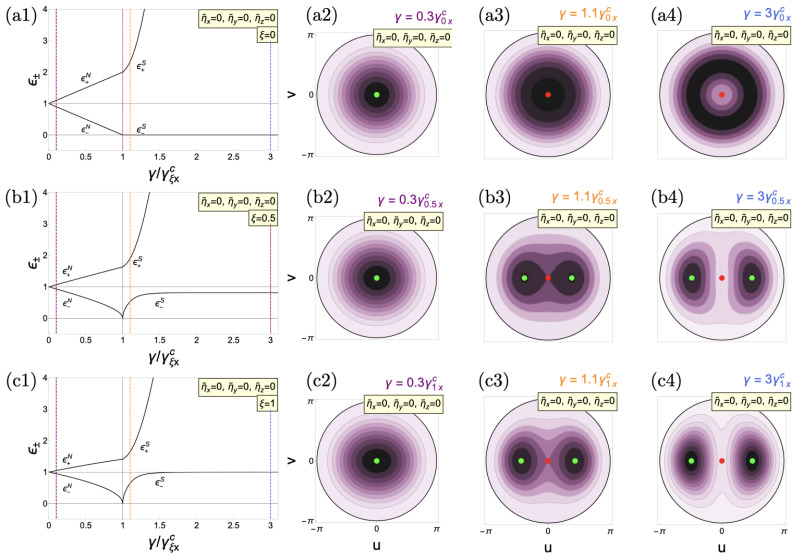
Polariton modes of the anisotropic Dicke model without material collective interactions. (**a1**) TC limit ( ξ=0 ), (**b1**) anisotropic case ( ξ=0.5 ), and (**c1**) Dicke limit ( ξ=1 ). The critical coupling $\gamma_{\xi x}^{c}$ ( $\gamma_{\xi y}^{c}$ ) is indicated by the vertical solid black (dotted red) line. (**a2**–**a4**,**b2**–**b4**,**c2**–**c4**) depict the corresponding energy surfaces for the respective cases. The vertical dashed purple line shows the position of energy surfaces in the energy spectrum in the normal phases (**a2**–**c2**). The yellow one indicates the location of energy surfaces in the superradiant phase (**a3**–**c3**), while the blue line represents higher values of light–matter couplings (**a4**–**c4**). Green points in the energy surfaces represent energy minima, red ones indicate maxima and yellow points denote saddle points. Tilde variables are scaled to $\omega_{0}$ . All cases are calculated in resonance ( $\omega =\omega_{0}=1$ ).

As shown in Figure 1, first, we revisit the polariton modes without matter interactions. In Figure 1(a1), we show the polariton modes in the Tavis–Cummings limit. There, the lower curve corresponds to the phase mode $\epsilon_{-}^{N}$ , and the upper one to the amplitude mode $\epsilon_{+}^{N}$ . In the normal phase, the parametric evolution of both modes increases proportionally to the light–matter coupling. Their energy gap is just the Rabi splitting 2γ . This agrees with the overall behavior of the energy surface, which adopts a spherical well shape, a reflection of the conserved U(1) symmetry [see Figure 1(a2)]. The dashed, vertical line marks the critical coupling $\gamma_{0x}^{\left(c\right)}=\gamma_{0+}^{\left(c\right)}$ , separating the normal from the superradiant phases. There, the energy surface experiences a sudden change, taking the shape of a Mexican hat (see [Figure 1(a3,a4)]. Because of the onset of degeneration, we have spontaneous symmetry breaking. Once we enter the superradiant phase, the phase mode $\epsilon_{-}^{S}$ becomes a Goldstone mode with zero energy, depicted on the energy surface as the minimum energy ring of the Mexican hat [22,23,88,106,170,171]. Instead, the amplitude mode $\epsilon_{+}^{S}$ depends on the brim of the Mexican hat. As it grows, it requires increasingly higher energy costs than the phase modes, which remain constant. Note that in this case, $\gamma_{0x}=\gamma_{0y}=\gamma_{0-}$ , so there is no presence of the superradiant- (−) effects. In polariton terms, the two modes represent a maximal light–matter hybridization in resonance ( $\omega =\omega_{0}$ ). The amplitude mode, being massive, can be identified as an upper-polariton branch that contains the most matter content, while the phase mode, being massless, becomes the lower-polariton branch with the most photonic content. Here, entering the superradiant phase implies a vanishment of the lower-polariton energy.

The energy spectra on Figure 1(b1,c1) correspond to the standard Dicke model (ξ=1) and the anisotropic case when ξ=0.5 , respectively. Both cases exhibit quite similar behavior. In the normal phase, the behavior resembles the TC limit because the system is in the strong-interacting regime. At the critical coupling $\gamma_{\xi x}^{c}=\gamma_{\xi +}^{c}$ , however, the phase mode tends to zero, and the amplitude mode to a fixed value given by

(44) $$\begin{array}{c} \epsilon_{+}^{N,c}=\sqrt{\frac{1}{2}\left(\omega^{2}+\omega_{0}^{2}\omega_{zx}^{2}\right)\left[\left(1+{\tilde{\omega }}_{zy}^{2}\right)+\sqrt{{\left(1-{\tilde{\omega }}_{zy}^{2}\right)}^{2}+4{\tilde{\omega }}_{zx}^{2}\frac{{\left(1-\xi \right)}^{2}}{{\left(1+\xi \right)}^{2}}}\right]}, \end{array}$$

in the normal phase, and by

(45) $$\begin{array}{c} \epsilon_{+}^{S,c}=\sqrt{\frac{1}{2}\left[\omega^{2}+\left(1-\frac{\eta_{z}}{\omega_{0}}\right)\omega_{0}^{2}{\tilde{\omega }}_{zx}^{2}\right]\left[\left(1+\frac{{\tilde{\omega }}_{zy}^{2}}{1-\frac{\eta_{z}}{\omega_{0}}}\right)+\sqrt{{\left(1-\frac{{\tilde{\omega }}_{zy}^{2}}{1-\frac{\eta_{z}}{\omega_{0}}}\right)}^{2}+\frac{4{\tilde{\omega }}_{zx}^{2}}{1-\frac{\eta_{z}}{\omega_{0}}}\frac{{\left(1-\xi \right)}^{2}}{{\left(1+\xi \right)}^{2}}}\right]} \end{array}$$

in the superradiant phase. Discontinuities may arise for $\eta_{z}\neq 0$ at the critical coupling. In the absence of interactions it becomes

(46) $$\begin{array}{c} \epsilon_{+}^{c}=\sqrt{\left(\omega^{2}+\omega_{0}^{2}\right)\left(1+\left|\frac{1-\xi }{1+\xi }\right|\right)}, \end{array}$$

and for the standard Dicke model, it is $\epsilon_{+}^{c}={\left(\omega^{2}+\omega_{0}^{2}\right)}^{1/2}$ [80]. When one passes the QPT, the phase mode gains a finite energy, becoming the rotonic mode [23,88,170], which converges to the finite value

(47) $$\begin{array}{c} \epsilon_{-}^{\gamma \gg 1}=\omega \sqrt{1+\left|\frac{1-\xi }{1+\xi }\right|}, \end{array}$$

for larger couplings. This value comes from the energy cost to pass between the two degenerate minima (whose phases are 0 and π ) observed in Figure 1(b3,b4). The amplitude mode behaves quite similarly to the TC since the center of the energy surface presents an unstable point and tends quadratically as a function of γ , $\epsilon_{+}^{\gamma \gg 1}={\left(1+\xi \right)}^{2}\gamma^{2}/\omega$ in the absence of matter interactions, which has the value $4\gamma^{2}/\omega$ in the standard Dicke model [80].

It has been shown that increasing the anisotropy decreases the amplitude mode gap until one obtains the Goldstone mode. This can be seen from Equation (47), where the limit ξ→0 leads to ω returning to the non-interacting value of photon field for the isotropic model [80]. This critical feature has been proposed as an experimental signature for detecting the Goldstone mode [18,22]. We observe this feature in Figure 1(c1), and it can be explained in terms of the change in the energy surface that leads to a new set of extremal points related to the superradiant- (−) phase that reduces the energy of the phase mode [122]. In this case, the presence of the counter-rotating terms that do not conserve the excitation number is reflected in the finite energy of the phase mode as a lower-polariton mode, where light gains “mass” close to the QPT and then becomes a massless but highly correlated state. In polariton terms, the lower-polariton energy becomes gapped. We notice, however, that the criticality of these cases is not affected by the superradiant- (−) extreme points because, in the absence of interactions, they cannot be shifted as it happens below. For the Dicke model $\gamma_{1-}^{c}\to \infty$ , and for the anisotropic case with ξ=0.5 , the critical coupling $\gamma_{0.5-}^{c}=3\gamma_{0.5+}^{c}$ is deep in the superradiant phase.

### 4.2. Presence of Matter–Matter Interactions

Next, we explore the effect of matter interactions over the polariton modes. In general, the *z*-interactions alone have the role of shifting both the critical coupling and the energy spectrum. To simplify the analysis, we will focus on the role of the *x* and *y* interactions via the relevant parameters $\Delta \eta_{zx}$ and $\Delta \eta_{zy}$ , respectively.

**Figure 2:**
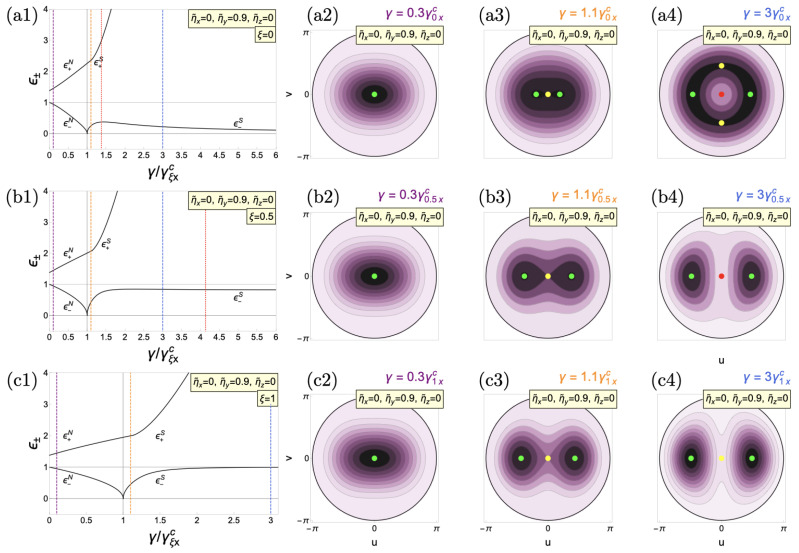
Same as Figure 1 but with material collective interactions at $\eta_{y}=0.9\omega_{0}$ ( $\Delta \eta_{zx}=0$ , $\Delta \eta_{zy}=-0.9\omega_{0}$ ).

In Figure 2, we show a representative value of the interactions given by $\eta_{y}=0.9\omega_{0}$ ( $\Delta \eta_{zy}=-0.9\omega_{0}$ ), keeping $\eta_{x}=0$ . Because of the interactions, in the normal phase, the energy surface widens along the *u* direction for all ξ , so we call it the *deformed normal* phase [122] [See Figure 2(a2–c2)]. In this case, the deformation does not substantially affect the phase and amplitude modes in the normal phase, except for an energy gap of the amplitude mode for zero spin–boson coupling given by

(48) $$\begin{array}{c} \epsilon_{+}^{N}=\omega_{0}{\tilde{\omega }}_{zx}{\tilde{\omega }}_{zy}. \end{array}$$

and representing the cost to climb the well in the presence of interactions. Next, we cross the superradiant QPT. For the three cases under study ( ξ=0,0.5,1 ), the amplitude mode is not significantly changed by the interactions compared to the case without. However, this is not the case for the phase mode in the TC limit ( ξ=0 ). As the system enters the superradiant phase, the excitation number is not conserved anymore because of the deformation, so we do not have the Mexican hat potential anymore. As shown in Figure 2(a1), the phase mode gains energy, becoming rotonic. As we increase coupling, the onset of new extreme points at higher energies contributes to somehow restoring the original symmetry of the Mexican hat potential in the absence of interactions and reducing the mode’s energy. Thus, as the spin–boson coupling increases, the phase mode slowly tends to a Goldstone-like phase mode. As a result, interactions produce a “variable” mass roton mode. In both the anisotropic and Dicke limit cases, the results are similar to the case without interactions. After transitioning to the superradiant phase, two stable degenerate minimum points form, so there is no mechanism capable of decreasing the energy of the phase mode and eventually converging to a general energy value given by Equation (47).

**Figure 3:**
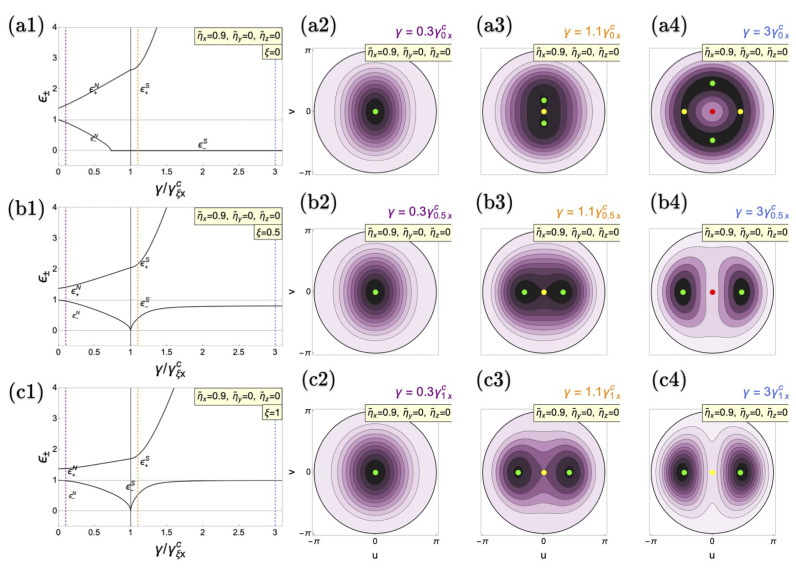
Polariton modes of the anisotropic Dicke model as a function of the coupling for (**a1**) TC limit ( ξ=0 ), (**b1**) anisotropic case ( ξ=0.5 ), and (**c1**) Dicke limit ( ξ=1 ) with material collective interactions at $\eta_{x}=0.9\omega_{0}$ ( $\Delta \eta_{zy}=0.0$ , $\Delta \eta_{zx}=-0.9\omega_{0}$ ). The critical coupling $\gamma_{\xi x}^{c}$ ( $\gamma_{\xi y}^{c}$ ) is indicated by the vertical solid black (dotted red) line. (**a2**–**a4**,**b2**–**b4**,**c2**–**c4**) depict the corresponding energy surfaces for the respective cases. The vertical dashed purple line shows the position of energy surfaces in the energy spectrum in the normal phases (**a2**–**c2**). The yellow one indicates the location of energy surfaces in the superradiant phase (**a3**–**c3**), while the blue line represents higher values of light–matter couplings (**a4**–**c4**). Green points in the energy surfaces represent energy minima, red ones indicate maxima and yellow points denote saddle points. Tilde variables are scaled to $\omega_{0}$ . All cases are calculated in resonance ( $\omega =\omega_{0}=1$ ).

The situation is different for *x* interactions as shown in Figure 3 for a representative value $\eta_{x}=0.9$ ( $\Delta \eta_{zx}=-0.9$ ). We observe similar dynamics for the upper- and lower-polariton modes to the previous case. However, new phenomena emerge from the interplay between the anisotropy and matter interactions, i.e., the ability of the latter to shift the position of the superradiant- (−) phenomena. Again, the normal phase becomes deformed for every ξ but now with a different orientation, alongside the *v* direction corresponding to the change from *y* to *x* interactions [122] [See Figure 3(a2–c2)]. While the overall behavior of the amplitude mode remains the same, in the TC limit [Figure 3(a1)], we observe that the phase mode vanishes in the normal phase before the onset of the critical coupling. This is not a Goldstone mode but a suppression due to matter–matter interactions. As it can be seen from Equation (19), two conditions make the phase mode vanish, either $f_{\xi x}=1$ or $g_{\xi y}=1$ . As it can be shown in Figure 3(a1), the relative position of the critical coupling $\gamma_{0x}^{c}$ with respect to $\gamma_{0y}^{c}$ is shifted thanks to $\eta_{x}\neq 0$ , creating a coupling regime where the phase mode cannot exist, although the amplitude mode does. This can be seen from the relation between the two critical couplings

(49) $$\begin{array}{c} \Delta \gamma =\gamma_{\xi x}^{c}-\gamma_{\xi y}^{c}=\gamma_{\xi x}^{c}\left(1-\frac{{\tilde{\omega }}_{zy}}{{\tilde{\omega }}_{zx}}\frac{1+\xi }{1-\xi }\right). \end{array}$$

and it occurs when Δγ<0 . This unique effect results from the influence of the matter interactions over the competence between the superradiant-+ and superradiant-*y* phases resulting from the anisotropy. This behavior does not occur in the Dicke and anisotropic cases [See Figure 3(a1,c1)], provided the onset of the superradiant-*y* behavior occurs deep in the superradiant-*x* one. However, for the anisotropic case, the interplay between the anisotropy and the matter interactions might shift the critical coupling $\gamma_{\xi y}^{c}$ to suppress the mode. Beyond that, for the Dicke and anisotropic cases, the behavior of the polariton modes remains qualitatively similar to previous cases, except for the π rotation of the energy surfaces as one crosses the superradiant QPT as it can be seen in Figure 3(b2,b3), as well as in Figure 3(c2,c3).

## 5. Geometric Phase in Presence of Matter Interactions

Next, we investigate the influence of matter interactions over the Berry or geometric phase induced by the bosonic field and the collective pseudospin over both polariton modes. The geometric phase is a topological quantum phase that the eigenstates of a Hamiltonian acquire when one goes adiabatically over a close path in the system’s parameter space, accounting for the Hilbert space geometry [129,130]. The geometric phase has already been studied for the Dicke model [137,138], extensions [140,141], and under the presence of matter interactions in the *z*-direction [142]. Mainly because it becomes singular at criticality, it has been shown to serve as a signature for the superradiant QPT. Also, matter interactions could modify its scaling behavior [142].

First, for comparison, we obtain the approximated ground-state energy without excitations. According to Equation (3), the scaled energy reads

(50) $$\begin{array}{c} \epsilon_{0}=E_{0}\left(\alpha ,\beta \right)/2j=\omega \alpha^{2}+\omega_{0}\beta^{2}-\frac{\omega_{0}}{2}-\gamma \sqrt{k}2\alpha \beta \left(1+\xi \right)+\eta_{x}k\beta^{2}+\eta_{z}{\left(\beta^{2}-\frac{1}{2}\right)}^{2}. \end{array}$$

It agrees with that calculated using coherent states as trial states [122]. Then, by substituting Equation (29) into Equation (50), we obtain the ground-state energy for both the normal and superradiant phases

(51) $$\begin{array}{c} \epsilon_{0}=\langle \Psi_{0}|2jH_{0}|\Psi_{0}\rangle =-\frac{j\omega_{0}}{2}\left\{\begin{array}{cc} 1-\frac{\eta_{z}}{2\omega_{0}} & \gamma <\gamma_{\xi x}^{c}\\ \frac{1}{2}\left(\mu_{x}+\frac{1}{\mu_{x}}\right)-\frac{\eta_{z}}{2\omega_{0}} & \gamma >\gamma_{\xi x}^{c} \end{array}\right. \end{array}$$

where $|\Psi_{0}\rangle$ is the ground-state of the system, and $\mu_{x}\left(\Delta \eta_{zx},\xi \right)={\left(\Delta \eta_{zx}/\omega_{0}+f_{\xi +}\right)}^{-1}$ , with $f_{\xi +}=\gamma^{2}/\gamma_{\xi +}^{2}$ . The ground-state energy signals the superradiant QPT modified by the anisotropy and matter interactions at $\gamma_{\xi x}^{c}$ ( $\mu_{x}=1$ ).

To calculate the geometric phase, we follow the standard procedure [138,139,172,173,174,175], where one considers a unitary transformation producing a cyclic trajectory over the parameter space. It may be an arbitrary adiabatic circulation generated by the photon number [140] or over the Bloch sphere [175]. First, let us consider an adiabatic circulation generated by the photon number

(52) $$\begin{array}{c} \hat{U}\left(\phi_{n}\right)=\exp\left(-i\phi_{n}\hat{n}\right)=\exp\left(-i\phi_{n}{\hat{a}}^{†}\hat{a}\right). \end{array}$$

It provides an additional phase to the bosonic annihilation (creation) operator $\hat{a}\to \hat{a}e^{i\phi_{n}}$ ( ${\hat{a}}^{†}\to {\hat{a}}^{†}e^{-i\phi_{n}}$ ), effectively displacing them. Next, we introduce a time-dependent unitary transformation, where the parameter $\phi_{n}\left(t\right)=\omega_{n}t$ varies adiabatically in the interval [0,2π) with angular frequency $\omega_{n}$ . The geometric phase is calculated in terms of the circuit integral of the Berry connection

(53) $$\begin{array}{c} \Gamma_{n}=i\int_{0}^{2\pi }\;d\phi_{n}\;\langle \Psi \left(\phi_{n}\right)|\frac{d}{d\phi_{n}}|\Psi \left(\phi_{n}\right)\rangle =2\pi \langle \Psi_{0}|\hat{n}|\Psi_{0}\rangle , \end{array}$$

where $\langle \Psi_{0}|\hat{n}|\Psi_{0}{\rangle =|\alpha |}^{2}$ is just the expectation value of the photon number within the HPA. Hence, the geometric phase reads

(54) $$\begin{array}{c} \Gamma_{n}=j\left\{\begin{array}{cc} 0 & \gamma <\gamma_{\xi x}^{c}\\ \frac{\pi }{2}{\left(\frac{\gamma }{\gamma_{\xi x}^{c}}\right)}^{2}\frac{\omega_{zx}^{2}}{\omega_{0}}\left(1-\mu_{x}^{2}\right) & \gamma >\gamma_{\xi x}^{c} \end{array}\right. \end{array}$$

For ξ=1 , we recover the results of the standard [138] and modified Dicke models [142]. Similarly, if now we consider a circulation generated by the excited pseudospin population ${\hat{J}}_{z}-jI$

(55) $$\begin{array}{c} \hat{V}\left(\phi_{m}\right)=\exp\left[-i\phi_{m}\left({\hat{J}}_{z}-jI\right)\right]=\exp\left(-i\phi_{m}{\hat{b}}^{†}\hat{b}\right). \end{array}$$

Like in the boson case, this shifts the bosonic annihilation (creation) operator $\hat{b}\to \hat{b}e^{i\phi_{m}}$ ( ${\hat{b}}^{†}\to {\hat{b}}^{†}e^{-i\phi_{m}}$ ), effectively displacing them. Changing adiabatically in time allows us to calculate the geometric phase as

(56) $$\begin{array}{c} \Gamma_{m}=i\int_{0}^{2\pi }\;d\phi_{m}\;\langle \Psi \left(\phi_{m}\right)|\frac{d}{d\phi_{m}}|\Psi \left(\phi_{m}\right)\rangle =2\pi \langle \Psi_{0}|{\hat{J}}_{z}-jI|\Psi_{0}\rangle , \end{array}$$

where $\langle \Psi_{0}|{\hat{J}}_{z}-jI|\Psi_{0}{\rangle =|\beta |}^{2}$ , so

(57) $$\begin{array}{c} \Gamma_{m}=j\left\{\begin{array}{cc} 0 & \gamma <\gamma_{\xi x}^{c}\\ \pi \left(1-\mu_{x}\right) & \gamma >\gamma_{\xi x}^{c} \end{array}\right. \end{array}$$

**Figure 4:**
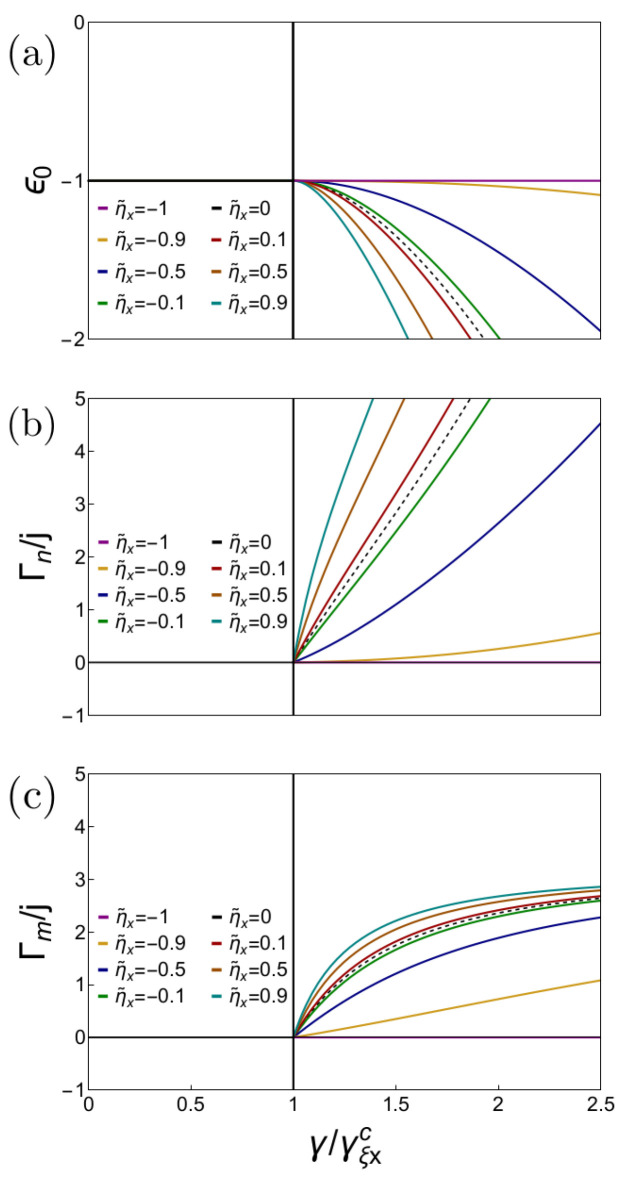
(**a**) Scaled ground-state energy $\epsilon_{0}=E_{0}/2j$ as a function of the light–matter coupling. (**b**) Scaled geometric phase $\Gamma_{n}$ considering an adiabatic circulation generated by the photon number. (**c**) Scaled geometric phase $\Gamma_{m}$ considering an adiabatic circulation generated by the collective pseudospin *z* projection. As indicated in the figures, we consider different values of the matter interactions $\Delta \eta_{zx}$ with $\eta_{z}=0$ . The case with $\Delta \eta_{zx}=0$ is indicated as a dashed line. All cases are calculated in resonance with $\omega =\omega_{0}=1$ .

We notice that, for these contours, both the $\Gamma_{n}$ and $\Gamma_{m}$ geometric phases depend only on the ratio $f_{\xi x}$ as in Equation (30), where $\mu_{x}^{-1}={\tilde{\omega }}_{zx}/\omega_{0}\left(f_{\xi x}-1\right)+1$ . As a result, the behavior is qualitatively independent from ξ and $\eta_{y}$ . That it does not depend on $\eta_{y}$ is because the extreme points in the energy surface belonging to the superradiant-*y* occur higher in energy, far away from the ground-state energy. Instead, the independence from ξ is a feature that captures the universal behavior of the superradiant QPT. In Figure 4, we show the results for both geometric phases $\Gamma_{n}$ and $\Gamma_{m}$ and compare them to the scaled ground-state energy. We observe that the geometric phase identifies the position of the superradiant QPT as expected. The geometric phases are sensitive to the sign of $\eta_{x}$ , increasing with $\eta_{x}$ . Furthermore, we observe that the geometric phase also helps to identify the first-order phase transition occurring at $\Delta \eta_{zx}=\omega_{0}$ , where the geometric phase becomes zero independently of γ in both cases and then turns negative.

## 6. Discussion and Conclusions

We derived analytic expressions for low-energy excitations of the anisotropic Dicke model in the presence of matter interactions by employing the Holstein–Primakoff transformation and neglecting higher-order terms in the thermodynamic limit. We explored the modes in the normal (ordered) phase, deformed by matter interactions, and the superradiant (ordered) phase from a unified point of view, whose interpretation is supported by the analysis of energy surfaces obtained using the corresponding classical Dicke model via coherent states [122].

The anisotropy in the Dicke model that, in this work, tunes the Hamiltonian between the Tavis–Cummings to the standard Dicke models produces a new set of extreme points and quantum phases, the superradiant- (+) and (−) phases, where superradiance is determined by the (+) phase, given that it lies lower in energy. Matter interactions serve two purposes: to shift the values of critical couplings and ground-state energy and to produce new extreme points in the energy surface, reflecting the onset of new quantum phases. Particularly, interactions in the *x* and *y* directions directly modify the criticality of the (+) and (−) phases, respectively, and shift their relative position in terms of the spin–boson coupling.

Beyond the shifting of critical couplings and ground-state energy associated to matter–matter interactions, we found unique effects from combining them with the anisotropy. In most cases, amplitude, massive, or upper-polariton mode behave qualitatively the same, just changing energetically to reflect the deformations of the energy surface, so interactions increase the gap to the phase mode, creating an energy cost given by $\omega_{0}{\tilde{\omega }}_{zx}{\tilde{\omega }}_{zy}$ . Novel behavior appears mostly over the ground-state mode, i.e., the phase or lower-polariton mode. For the Dicke model, it becomes a roton mode that, for larger couplings, tends to the non-interacting photon energy ω and decreases in energy with the anisotropy. Without interactions, it eventually transforms into the Goldstone mode. Instead, the TC limit exhibits a Goldstone mode in the absence of matter–matter interactions that becomes rotonic as the latter breaks the U(2) symmetry, leaving conserved only the $Z_{2}$ one.

A major result is that tuning the combination of matter interactions in the *z* and *y* directions can reduce the rotonic mode energy in the TC Hamiltonian, producing a “variable” mass roton mode, or a lower polariton that starts losing matter content with interactions. This is understood in terms of the energy surface, as the interactions’ influence on higher energies becomes less relevant to the ground state, thus restoring to some extent the Goldstone-like behavior. Second, we discover that the Goldstone mode can be suppressed by tuning the interactions in the *z* and *x* directions by shifting the relative position between the critical couplings of the superradian-*x* and -*y* phases. This suppression does not affect the amplitude mode.

We anticipate these features could be detected and employed as an experimental signature to observe the Goldstone mode in novel setups, such as spin–magnon systems, where the interactions are tunable [109,110,111], as well as in others within the broad range of setups where the Dicke model can be realized, from quantum optics and atomic physics to condensed matter.

We also calculated the geometric phase for both boson and pseudospin contours, which equals the expectation value of the photon number and excited pseudospin in the phase mode within this approximation. We found that the role of the *z* and *x* interactions is to shift the phase. Still, it is independent of the *y* interactions and the anisotropy parameter ξ , as it is a low-energy approach capable only of capturing the universal behavior of the superradiant-*x* transition. Furthermore, the geometric phase becomes singular at the superradiant QPT, serving as a signature of criticality, where we recover previous results from refs. [119,142]. It also detects the first-order QPT that results from changing matter–matter interactions by changing sign.

We conjecture that the predominance of the superradiant-*x* phase will be inverted to the superradiant-*y* phase in the case of the anti-Tavis–Cummings model, where now the rotating terms become smaller than the counter-rotating ones [18,92,176]. Our model can be extended to explain this case, but this goes beyond the scope of this work. Finally, we note that establishing a quantitative comparison of the polariton modes with finite-size quantum exact numerical solutions as a function of energy is desirable [150]. Nevertheless, this analysis and exploring the effect of the anisotropy and matter interactions over the polariton modes beyond the mean field are left for future work [82,177].

## Data Availability

Dataset available on request from the authors.

## Abbreviations

The following abbreviations are used in this manuscript:

AH	Anderson–Higgs
HPT	Holstein–Primakoff Transformation
LMG	Lipking–Meshkov–Glick
NG	Nambu–Goldstone
QPT	Quantum Phase Transition
TC	Tavis–Cummings

## References

[1] Anderson P.W. (1963). Plasmons, Gauge Invariance, and Mass. Phys. Rev..

[2] Higgs P.W. (1964). Broken Symmetries and the Masses of Gauge Bosons. Phys. Rev. Lett..

[3] Goldstone J. (1961). Field theories with “Superconductor” solutions. Nuovo C. (1955–1965).

[4] Nambu Y., Jona-Lasinio G. (1961). Dynamical Model of Elementary Particles Based on an Analogy with Superconductivity. II. Phys. Rev..

[5] Littlewood P.B., Varma C.M. (1981). Gauge-Invariant Theory of the Dynamical Interaction of Charge Density Waves and Superconductivity. Phys. Rev. Lett..

[6] Littlewood P.B., Varma C.M. (1982). Amplitude collective modes in superconductors and their coupling to charge-density waves. Phys. Rev. B.

[7] Varma C.M. (2002). Higgs Boson in Superconductors. J. Low Temp. Phys..

[8] Pekker D., Varma C. (2015). Amplitude/Higgs Modes in Condensed Matter Physics. Annu. Rev. Condens. Matter Phys..

[9] Méasson M.A., Gallais Y., Cazayous M., Clair B., Rodière P., Cario L., Sacuto A. (2014). Amplitude Higgs mode in the 2*H*-NbSe_2_ superconductor. Phys. Rev. B.

[10] Chubukov A.V., Sachdev S., Ye J. (1994). Theory of two-dimensional quantum Heisenberg antiferromagnets with a nearly critical ground state. Phys. Rev. B.

[11] Podolsky D., Sachdev S. (2012). Spectral functions of the Higgs mode near two-dimensional quantum critical points. Phys. Rev. B.

[12] Rüegg C., Normand B., Matsumoto M., Furrer A., McMorrow D.F., Krämer K.W., Güdel H.U., Gvasaliya S.N., Mutka H., Boehm M. (2008). Quantum Magnets under Pressure: Controlling Elementary Excitations in *TlCuCl*_3_. Phys. Rev. Lett..

[13] Englert F., Brout R. (1964). Broken Symmetry and the Mass of Gauge Vector Mesons. Phys. Rev. Lett..

[14] Guralnik G.S., Hagen C.R., Kibble T.W.B. (1964). Global Conservation Laws and Massless Particles. Phys. Rev. Lett..

[15] Bernstein J. (1974). Spontaneous symmetry breaking, gauge theories, the Higgs mechanism and all that. Rev. Mod. Phys..

[16] Sachdev S. (1999). Quantum Phase Transitions.

[17] Carr L.D. (2010). Understanding Quantum Phase Transitions.

[18] Baksic A., Ciuti C. (2014). Controlling Discrete and Continuous Symmetries in “Superradiant” Phase Transitions with Circuit QED Systems. Phys. Rev. Lett..

[19] Yi-Xiang Y., Ye J., Liu W.M. (2013). Goldstone and Higgs modes of photons inside a cavity. Sci. Rep..

[20] Bissbort U., Götze S., Li Y., Heinze J., Krauser J.S., Weinberg M., Becker C., Sengstock K., Hofstetter W. (2011). Detecting the Amplitude Mode of Strongly Interacting Lattice Bosons by Bragg Scattering. Phys. Rev. Lett..

[21] Endres M., Fukuhara T., Pekker D., Cheneau M., Schauβ P., Gross C., Demler E., Kuhr S., Bloch I. (2012). The ‘Higgs’ amplitude mode at the two-dimensional superfluid/Mott insulator transition. Nature.

[22] Léonard J., Morales A., Zupancic P., Donner T., Esslinger T. (2017). Monitoring and manipulating Higgs and Goldstone modes in a supersolid quantum gas. Science.

[23] Chiacchio E.I.R., Nunnenkamp A. (2018). Emergence of continuous rotational symmetries in ultracold atoms coupled to optical cavities. Phys. Rev. A.

[24] Schuster S.C., Wolf P., Ostermann S., Slama S., Zimmermann C. (2020). Supersolid Properties of a Bose-Einstein Condensate in a Ring Resonator. Phys. Rev. Lett..

[25] Hepp K., Lieb E.H. (1973). On the superradiant phase transition for molecules in a quantized radiation field: The dicke maser model. Ann. Phys..

[26] Wang Y.K., Hioe F.T. (1973). Phase Transition in the Dicke Model of Superradiance. Phys. Rev. A.

[27] Larson J., Irish E.K. (2017). Some remarks on ‘superradiant’ phase transitions in light-matter systems. J. Phys. A Math. Theor..

[28] Dicke R.H. (1954). Coherence in Spontaneous Radiation Processes. Phys. Rev..

[29] Garraway B.M. (2011). The Dicke model in quantum optics: Dicke model revisited. Philos. Trans. R. Soc. A Math. Phys. Eng. Sci..

[30] Kirton P., Roses M.M., Keeling J., Dalla Torre E.G. (2019). Introduction to the Dicke Model: From Equilibrium to Nonequilibrium, and Vice Versa. Adv. Quantum Technol..

[31] Le Boité A. (2020). Theoretical Methods for Ultrastrong Light–Matter Interactions. Adv. Quantum Technol..

[32] Larson J., Mavrogordatos T. (2021). The Jaynes-Cummings Model and Its Descendants.

[33] Lambert N., Emary C., Brandes T. (2004). Entanglement and the Phase Transition in Single-Mode Superradiance. Phys. Rev. Lett..

[34] Brandes T. (2005). Coherent and collective quantum optical effects in mesoscopic systems. Phys. Rep..

[35] Vidal J., Dusuel S. (2006). Finite-size scaling exponents in the Dicke model. EPL Europhys. Lett..

[36] Villaseñor D., Pilatowsky-Cameo S., Bastarrachea-Magnani M.A., Lerma-Hernández S., Santos L.F., Hirsch J.G. (2023). Chaos and Thermalization in the Spin-Boson Dicke Model. Entropy.

[37] de Aguiar M.A.M., Furuya K., Lewenkopf C.H., Nemes M.C. (1991). Particle-Spin Coupling in a Chaotic System: Localization-Delocalization in the Husimi Distributions. EPL Europhys. Lett..

[38] de Aguiar M., Furuya K., Lewenkopf C., Nemes M. (1992). Chaos in a spin-boson system: Classical analysis. Ann. Phys..

[39] Furuya K., de Aguiar M., Lewenkopf C., Nemes M. (1992). Husimi distributions of a spin-boson system and the signatures of its classical dynamics. Ann. Phys..

[40] Bastarrachea-Magnani M.A., López-del-Carpio B., Lerma-Hernández S., Hirsch J.G. (2015). Chaos in the Dicke model: Quantum and semiclassical analysis. Phys. Scr..

[41] Pérez-Fernández P., Relaño A., Arias J.M., Cejnar P., Dukelsky J., García-Ramos J.E. (2011). Excited-state phase transition and onset of chaos in quantum optical models. Phys. Rev. E.

[42] Stránský P., Macek M., Cejnar P. (2014). Excited-state quantum phase transitions in systems with two degrees of freedom: Level density, level dynamics, thermal properties. Ann. Phys..

[43] Stránský P., Macek M., Leviatan A., Cejnar P. (2015). Excited-state quantum phase transitions in systems with two degrees of freedom: II. Finite-size effects. Ann. Phys..

[44] Cejnar P., Stránský P., Macek M., Kloc M. (2021). Excited-state quantum phase transitions. J. Phys. A Math. Theor..

[45] Bastidas V.M., Emary C., Regler B., Brandes T. (2012). Nonequilibrium Quantum Phase Transitions in the Dicke Model. Phys. Rev. Lett..

[46] Kloc M., Stránský P., Cejnar P. (2018). Quantum quench dynamics in Dicke superradiance models. Phys. Rev. A.

[47] Shen L., Shi Z., Yang Z., Wu H., Zhong Z., Zheng S. (2020). A similarity of quantum phase transition and quench dynamics in the Dicke model beyond the thermodynamic limit. EPJ Quantum Technol..

[48] Forn-Díaz P., Lamata L., Rico E., Kono J., Solano E. (2019). Ultrastrong coupling regimes of light-matter interaction. Rev. Mod. Phys..

[49] Frisk Kockum A., Miranowicz A., De Liberato S., Savasta S., Nori F. (2019). Ultrastrong coupling between light and matter. Nat. Rev. Phys..

[50] Peraca N.M., Baydin A., Gao W., Bamba M., Kono J., Cundiff S.T., Kira M. (2020). Chapter Three—Ultrastrong light–matter coupling in semiconductors. Semiconductor Quantum Science and Technology.

[51] Nagy D., Kónya G., Szirmai G., Domokos P. (2010). Dicke-Model Phase Transition in the Quantum Motion of a Bose-Einstein Condensate in an Optical Cavity. Phys. Rev. Lett..

[52] Liu N., Lian J., Ma J., Xiao L., Chen G., Liang J.Q., Jia S. (2011). Light-shift-induced quantum phase transitions of a Bose-Einstein condensate in an optical cavity. Phys. Rev. A.

[53] Yuan J.B., Lu W.J., Song Y.J., Kuang L.M. (2017). Single-impurity-induced Dicke quantum phase transition in a cavity-Bose-Einstein condensate. Sci. Rep..

[54] Jaako T., Xiang Z.L., Garcia-Ripoll J.J., Rabl P. (2016). Ultrastrong-coupling phenomena beyond the Dicke model. Phys. Rev. A.

[55] Yang W.J., Wang X.B. (2017). Ultrastrong-coupling quantum-phase-transition phenomena in a few-qubit circuit QED system. Phys. Rev. A.

[56] De Bernardis D., Jaako T., Rabl P. (2018). Cavity quantum electrodynamics in the nonperturbative regime. Phys. Rev. A.

[57] Pilar P., De Bernardis D., Rabl P. (2020). Thermodynamics of ultrastrongly coupled light-matter systems. Quantum.

[58] Auerbach N., Zelevinsky V. (2011). Super-radiant dynamics, doorways and resonances in nuclei and other open mesoscopic systems. Rep. Prog. Phys..

[59] Cong K., Zhang Q., Wang Y., Noe G.T., Belyanin A., Kono J. (2016). Dicke superradiance in solids. J. Opt. Soc. Am. B.

[60] Hagenmüller D., Ciuti C. (2012). Cavity QED of the Graphene Cyclotron Transition. Phys. Rev. Lett..

[61] Chirolli L., Polini M., Giovannetti V., MacDonald A.H. (2012). Drude Weight, Cyclotron Resonance, and the Dicke Model of Graphene Cavity QED. Phys. Rev. Lett..

[62] Scheibner M., Schmidt T., Worschech L., Forchel A., Bacher G., Passow T., Hommel D. (2007). Superradiance of quantum dots. Nat. Phys..

[63] Blais A., Huang R.S., Wallraff A., Girvin S.M., Schoelkopf R.J. (2004). Cavity quantum electrodynamics for superconducting electrical circuits: An architecture for quantum computation. Phys. Rev. A.

[64] Casanova J., Romero G., Lizuain I., García-Ripoll J.J., Solano E. (2010). Deep Strong Coupling Regime of the Jaynes-Cummings Model. Phys. Rev. Lett..

[65] Mezzacapo A., Las Heras U., Pedernales J.S., DiCarlo L., Solano E., Lamata L. (2014). Digital Quantum Rabi and Dicke Models in Superconducting Circuits. Sci. Rep..

[66] Dimer F., Estienne B., Parkins A.S., Carmichael H.J. (2007). Proposed realization of the Dicke-model quantum phase transition in an optical cavity QED system. Phys. Rev. A.

[67] Baden M.P., Arnold K.J., Grimsmo A.L., Parkins S., Barrett M.D. (2014). Realization of the Dicke Model Using Cavity-Assisted Raman Transitions. Phys. Rev. Lett..

[68] Schneble D., Torii Y., Boyd M., Streed E.W., Pritchard D.E., Ketterle W. (2003). The Onset of Matter-Wave Amplification in a Superradiant Bose-Einstein Condensate. Science.

[69] Baumann K., Guerlin C., Brennecke F., Esslinger T. (2010). Dicke quantum phase transition with a superfluid gas in an optical cavity. Nature.

[70] Baumann K., Mottl R., Brennecke F., Esslinger T. (2011). Exploring Symmetry Breaking at the Dicke Quantum Phase Transition. Phys. Rev. Lett..

[71] Klinder J., Keßler H., Wolke M., Mathey L., Hemmerich A. (2015). Dynamical phase transition in the open Dicke model. Proc. Natl. Acad. Sci. USA.

[72] Keeling J., Bhaseen M.J., Simons B.D. (2010). Collective Dynamics of Bose-Einstein Condensates in Optical Cavities. Phys. Rev. Lett..

[73] Zhang X., Chen Y., Wu Z., Wang J., Fan J., Deng S., Wu H. (2021). Observation of a superradiant quantum phase transition in an intracavity degenerate Fermi gas. Science.

[74] Helson V., Zwettler T., Mivehvar F., Colella E., Roux K., Konishi H., Ritsch H., Brantut J.P. (2023). Density-wave ordering in a unitary Fermi gas with photon-mediated interactions. Nature.

[75] Mivehvar F., Ostermann S., Piazza F., Ritsch H. (2018). Driven-Dissipative Supersolid in a Ring Cavity. Phys. Rev. Lett..

[76] Mivehvar F., Piazza F., Donner T., Ritsch H. (2021). Cavity QED with quantum gases: New paradigms in many-body physics. Adv. Phys..

[77] Tavis M., Cummings F.W. (1968). Exact Solution for an *N*-Molecule—Radiation-Field Hamiltonian. Phys. Rev..

[78] Bastarrachea-Magnani M.A., Lerma-Hernández S., Hirsch J.G. (2014). Comparative quantum and semiclassical analysis of atom-field systems. I. Density of states and excited-state quantum phase transitions. Phys. Rev. A.

[79] Emary C., Brandes T. (2003). Quantum Chaos Triggered by Precursors of a Quantum Phase Transition: The Dicke Model. Phys. Rev. Lett..

[80] Emary C., Brandes T. (2003). Chaos and the quantum phase transition in the Dicke model. Phys. Rev. E.

[81] Mottl R., Brennecke F., Baumann K., Landig R., Donner T., Esslinger T. (2012). Roton-Type Mode Softening in a Quantum Gas with Cavity-Mediated Long-Range Interactions. Science.

[82] Eastham P.R., Littlewood P.B. (2001). Bose condensation of cavity polaritons beyond the linear regime: The thermal equilibrium of a model microcavity. Phys. Rev. B.

[83] Hopfield J.J. (1958). Theory of the Contribution of Excitons to the Complex Dielectric Constant of Crystals. Phys. Rev..

[84] Carusotto I., Ciuti C. (2013). Quantum fluids of light. Rev. Mod. Phys..

[85] Ciuti C., Bastard G., Carusotto I. (2005). Quantum vacuum properties of the intersubband cavity polariton field. Phys. Rev. B.

[86] Bastarrachea-Magnani M.A., Hirsch J.G. (2011). Numerical solutions of the Dicke Hamiltonian. Rev. Mex. Fis. S.

[87] Liu T., Zhang Y.Y., Chen Q.H., Wang K.L. (2009). Large-*N* scaling behavior of the ground-state energy, fidelity, and the order parameter in the Dicke model. Phys. Rev. A.

[88] Fan J., Yang Z., Zhang Y., Ma J., Chen G., Jia S. (2014). Hidden continuous symmetry and Nambu-Goldstone mode in a two-mode Dicke model. Phys. Rev. A.

[89] Ivanov P.A., Singer K., Vitanov N.V., Porras D. (2015). Quantum Sensors Assisted by Spontaneous Symmetry Breaking for Detecting Very Small Forces. Phys. Rev. Appl..

[90] Buijsman W., Gritsev V., Sprik R. (2017). Nonergodicity in the Anisotropic Dicke Model. Phys. Rev. Lett..

[91] Liu M., Chesi S., Ying Z.J., Chen X., Luo H.G., Lin H.Q. (2017). Universal Scaling and Critical Exponents of the Anisotropic Quantum Rabi Model. Phys. Rev. Lett..

[92] Shapiro D.S., Pogosov W.V., Lozovik Y.E. (2020). Universal fluctuations and squeezing in a generalized Dicke model near the superradiant phase transition. Phys. Rev. A.

[93] Bastarrachea-Magnani M.A., Lerma-Hernández S., Hirsch J.G. (2016). Thermal and quantum phase transitions in atom-field systems: A microcanonical analysis. J. Stat. Mech. Theory Exp..

[94] Kloc M., Stránský P., Cejnar P. (2017). Quantum phases and entanglement properties of an extended Dicke model. Ann. Phys..

[95] Das P., Sharma A. (2022). Revisiting the phase transitions of the Dicke model. Phys. Rev. A.

[96] Das P., Bhakuni D.S., Sharma A. (2023). Phase transitions of the anisotropic Dicke model. Phys. Rev. A.

[97] Bhaseen M.J., Mayoh J., Simons B.D., Keeling J. (2012). Dynamics of nonequilibrium Dicke models. Phys. Rev. A.

[98] Ferri F., Rosa-Medina R., Finger F., Dogra N., Soriente M., Zilberberg O., Donner T., Esslinger T. (2021). Emerging Dissipative Phases in a Superradiant Quantum Gas with Tunable Decay. Phys. Rev. X.

[99] Soriente M., Donner T., Chitra R., Zilberberg O. (2018). Dissipation-Induced Anomalous Multicritical Phenomena. Phys. Rev. Lett..

[100] Stitely K.C., Giraldo A., Krauskopf B., Parkins S. (2020). Nonlinear semiclassical dynamics of the unbalanced, open Dicke model. Phys. Rev. Res..

[101] Emary C., Brandes T. (2004). Phase transitions in generalized spin-boson (Dicke) models. Phys. Rev. A.

[102] Ivanov P.A., Porras D., Ivanov S.S., Schmidt-Kaler F. (2013). Simulation of the Jahn–Teller–Dicke magnetic structural phase transition with trapped ions. J. Phys. B At. Mol. Opt. Phys..

[103] Hayn M., Emary C., Brandes T. (2011). Phase transitions and dark-state physics in two-color superradiance. Phys. Rev. A.

[104] Moodie R.I., Ballantine K.E., Keeling J. (2018). Generalized classes of continuous symmetries in two-mode Dicke models. Phys. Rev. A.

[105] Palacino R., Keeling J. (2021). Atom-only theories for U(1) symmetric cavity-QED models. Phys. Rev. Res..

[106] Hwang M.J., Plenio M.B. (2016). Quantum Phase Transition in the Finite Jaynes-Cummings Lattice Systems. Phys. Rev. Lett..

[107] Cordero S., Nahmad-Achar E., López-Peña R., Castaños O. (2021). Quantum phase diagrams of matter-field Hamiltonians I: Fidelity, Bures distance, and entanglement. Phys. Scr..

[108] López-Peña R., Cordero S., Nahmad-Achar E., Castaños O. (2021). Quantum phase diagrams of matter-field Hamiltonians II: Wigner function analysis. Phys. Scr..

[109] Li X., Bamba M., Yuan N., Zhang Q., Zhao Y., Xiang M., Xu K., Jin Z., Ren W., Ma G. (2018). Observation of Dicke cooperativity in magnetic interactions. Science.

[110] Bamba M., Li X., Marquez Peraca N., Kono J. (2022). Magnonic superradiant phase transition. Commun. Phys..

[111] Marquez Peraca N., Li X., Moya J.M., Hayashida K., Kim D., Ma X., Neubauer K.J., Fallas Padilla D., Huang C.L., Dai P. (2024). Quantum simulation of an extended Dicke model with a magnetic solid. Commun. Mater..

[112] Lee C.F., Johnson N.F. (2004). First-Order Superradiant Phase Transitions in a Multiqubit Cavity System. Phys. Rev. Lett..

[113] Chen G., Wang X., Liang J.Q., Wang Z.D. (2008). Exotic quantum phase transitions in a Bose-Einstein condensate coupled to an optical cavity. Phys. Rev. A.

[114] Chen Q.H., Liu T., Zhang Y.Y., Wang K.L. (2010). Quantum phase transitions in coupled two-level atoms in a single-mode cavity. Phys. Rev. A.

[115] Rodríguez-Lara B.M., Lee R.K. (2011). Classical dynamics of a two-species condensate driven by a quantum field. Phys. Rev. E.

[116] Zhao X.Q., Liu N., Liang J.Q. (2017). First-Order Quantum Phase Transition for Dicke Model Induced by Atom-Atom Interaction. Commun. Theor. Phys..

[117] Rodriguez J.P.J., Chilingaryan S.A., Rodríguez-Lara B.M. (2018). Critical phenomena in an extended Dicke model. Phys. Rev. A.

[118] Yang L.P., Jacob Z. (2019). Quantum critical detector: Amplifying weak signals using discontinuous quantum phase transitions. Opt. Express.

[119] Chen G., Zhao D., Chen Z. (2006). Quantum phase transition for the Dicke model with the dipole–dipole interactions. J. Phys. B At. Mol. Opt. Phys..

[120] Nie J., Huang X., Yi X. (2009). Critical properties of entanglement in the Dicke model with the dipole–dipole interactions. Opt. Commun..

[121] Robles Robles R.A., Chilingaryan S.A., Rodríguez-Lara B.M., Lee R.K. (2015). Ground state in the finite Dicke model for interacting qubits. Phys. Rev. A.

[122] Herrera Romero R., Bastarrachea-Magnani M.A., Linares R. (2022). Critical Phenomena in Light–Matter Systems with Collective Matter Interactions. Entropy.

[123] Liu W., Duan L. (2023). Quantum Phase Transitions in a Generalized Dicke Model. Entropy.

[124] Sinha S., Sinha S. (2020). Chaos and Quantum Scars in Bose-Josephson Junction Coupled to a Bosonic Mode. Phys. Rev. Lett..

[125] Wang Q. (2022). Quantum Chaos in the Extended Dicke Model. Entropy.

[126] Román-Roche J., Gómez-León A., Luis F., Zueco D. (2024). Cavity QED materials: Comparison and validation of two linear response theories at arbitrary light-matter coupling strengths. arXiv.

[127] Román-Roche J., Gómez-León A., Luis F., Zueco D. (2024). Linear response theory for cavity QED materials. arXiv.

[128] Hirsch J.G., Castaños O., López-Peña R., Nahmad-Achar E. (2013). Virtues and limitations of the truncated Holstein-Primakoff description of quantum rotors. Phys. Scr..

[129] Berry M.V. (1984). Quantal phase factors accompanying adiabatic changes. Proc. R. Soc. London. A. Math. Phys. Sci..

[130] Berry M.V. (1985). Classical adiabatic angles and quantal adiabatic phase. J. Phys. A Math. Gen..

[131] Carollo A.C.M., Pachos J.K. (2005). Geometric Phases and Criticality in Spin-Chain Systems. Phys. Rev. Lett..

[132] Pachos J.K., Carollo A.C. (2006). Geometric phases and criticality in spin systems. Philos. Trans. R. Soc. A Math. Phys. Eng. Sci..

[133] Reuter M.E., Hartmann M.J., Plenio M.B. (2007). Geometric phases and critical phenomena in a chain of interacting spins. Proc. R. Soc. A Math. Phys. Eng. Sci..

[134] Peng X., Wu S., Li J., Suter D., Du J. (2010). Observation of the Ground-State Geometric Phase in a Heisenberg *XY* Model. Phys. Rev. Lett..

[135] Leek P.J., Fink J.M., Blais A., Bianchetti R., Göppl M., Gambetta J.M., Schuster D.I., Frunzio L., Schoelkopf R.J., Wallraff A. (2007). Observation of Berry’s Phase in a Solid-State Qubit. Science.

[136] Zhang Z., Wang T., Xiang L., Yao J., Wu J., Yin Y. (2017). Measuring the Berry phase in a superconducting phase qubit by a shortcut to adiabaticity. Phys. Rev. A.

[137] Plastina F., Liberti G., Carollo A. (2006). Scaling of Berry’s phase close to the Dicke quantum phase transition. Europhys. Lett..

[138] Chen G., Li J., Liang J.Q. (2006). Critical property of the geometric phase in the Dicke model. Phys. Rev. A.

[139] Carollo A., Valenti D., Spagnolo B. (2020). Geometry of quantum phase transitions. Phys. Rep..

[140] Guerra C.A.E., Mahecha-Gómez J., Hirsch J.G. (2020). Quantum phase transition and Berry phase in an extended Dicke model. Eur. Phys. J. D.

[141] Lu W., Zhai C., Liu Y., Song Y., Yuan J., Tang S. (2022). Berry Phase of Two Impurity Qubits as a Signature of Dicke Quantum Phase Transition. Photonics.

[142] Li S.C., Liu H.L., Zhao X.Y. (2013). Quantum phase transition and geometric phase in a coupled cavity-BEC system. Eur. Phys. J. D.

[143] Chen G., Chen Z., Liang J.Q. (2007). Ground-state properties for coupled Bose-Einstein condensates inside a cavity quantum electrodynamics. Europhys. Lett. (EPL).

[144] Sinha S., Sinha S. (2019). Dissipative Bose-Josephson junction coupled to bosonic baths. Phys. Rev. E.

[145] Joshi A., Puri R.R., Lawande S.V. (1991). Effect of dipole interaction and phase-interrupting collisions on the collapse-and-revival phenomenon in the Jaynes-Cummings model. Phys. Rev. A.

[146] Abdel-Rady A.S., Hassan S.S.A., Osman A.N.A., Salah A. (2017). Evolution of Extended JC-Dicke Quantum Phase Transition with a Coupled Optical Cavity in Bose-Einstein Condensate System. Int. J. Theor. Phys..

[147] Salah A., Abdel-Rady A.S., Osman A.N.A., Hassan S.S.A. (2018). Enhancing quantum phase transitions in the critical point of Extended TC-Dicke model via Stark effect. Sci. Rep..

[148] Tian L. (2010). Circuit QED and Sudden Phase Switching in a Superconducting Qubit Array. Phys. Rev. Lett..

[149] Zhang Y., Yu L., Liang J.Q., Chen G., Jia S., Nori F. (2014). Quantum phases in circuit QED with a superconducting qubit array. Sci. Rep..

[150] Bastarrachea-Magnani M.A., Lerma-Hernández S., Hirsch J.G. (2014). Comparative quantum and semiclassical analysis of atom-field systems. II. Chaos and regularity. Phys. Rev. A.

[151] Chávez-Carlos J., Bastarrachea-Magnani M.A., Lerma-Hernández S., Hirsch J.G. (2016). Classical chaos in atom-field systems. Phys. Rev. E.

[152] Zhang W.M., Feng D.H., Gilmore R. (1990). Coherent states: Theory and some applications. Rev. Mod. Phys..

[153] Gilmore R. (1993). Catastrophe Theory for Scientists and Engineers.

[154] Castaños O., Nahmad-Achar E., López-Peña R., Hirsch J.G. (2011). Superradiant phase in field-matter interactions. Phys. Rev. A.

[155] Lipkin H.J., Meshkov N., Glick A. (1965). Validity of many-body approximation methods for a solvable model: (I). Exact solutions and perturbation theory. Nucl. Phys..

[156] Meshkov N., Glick A.J., Lipkin H.J. (1965). Validity of many-body approximation methods for a solvable model: (II). Linearization procedures. Nucl. Phys..

[157] Glick A.J., Lipkin H.J., Meshkov N. (1965). Validity of many-body approximation methods for a solvable model: (III). Diagram summations. Nucl. Phys..

[158] Dusuel S., Vidal J. (2004). Finite-Size Scaling Exponents of the Lipkin-Meshkov-Glick Model. Phys. Rev. Lett..

[159] Dusuel S., Vidal J. (2005). Continuous unitary transformations and finite-size scaling exponents in the Lipkin-Meshkov-Glick model. Phys. Rev. B.

[160] Castaños O., López-Peña R., Hirsch J.G., López-Moreno E. (2006). Classical and quantum phase transitions in the Lipkin-Meshkov-Glick model. Phys. Rev. B.

[161] Heiss W.D., Scholtz F.G., Geyer H.B. (2005). The large N behaviour of the Lipkin model and exceptional points. J. Phys. A Math. Gen..

[162] Leyvraz F., Heiss W.D. (2005). Large-*N* Scaling Behavior of the Lipkin-Meshkov-Glick Model. Phys. Rev. Lett..

[163] Heiss W.D. (2006). On the thermodynamic limit of the Lipkin model. J. Phys. A Math. Gen..

[164] Ribeiro P., Vidal J., Mosseri R. (2008). Exact spectrum of the Lipkin-Meshkov-Glick model in the thermodynamic limit and finite-size corrections. Phys. Rev. E.

[165] Engelhardt G., Bastidas V.M., Kopylov W., Brandes T. (2015). Excited-state quantum phase transitions and periodic dynamics. Phys. Rev. A.

[166] García-Ramos J.E., Pérez-Fernández P., Arias J.M. (2017). Excited-state quantum phase transitions in a two-fluid Lipkin model. Phys. Rev. C.

[167] Holstein T., Primakoff H. (1940). Field Dependence of the Intrinsic Domain Magnetization of a Ferromagnet. Phys. Rev..

[168] Bakemeier L., Alvermann A., Fehske H. (2013). Dynamics of the Dicke model close to the classical limit. Phys. Rev. A.

[169] Goldstein H., Poole C.P., Safko J. (2001). Classical Mechanics.

[170] Huang J.F., Tian L. (2023). Modulation-based superradiant phase transition in the strong-coupling regime. Phys. Rev. A.

[171] Deng Y., Yi S. (2023). Self-ordered supersolid phase beyond Dicke superradiance in a ring cavity. Phys. Rev. Res..

[172] Fuentes-Guridi I., Carollo A., Bose S., Vedral V. (2002). Vacuum Induced Spin-1/2 Berry’s Phase. Phys. Rev. Lett..

[173] Carollo A., França Santos M., Vedral V. (2003). Berry’s phase in cavity QED: Proposal for observing an effect of field quantization. Phys. Rev. A.

[174] Carollo A., Fuentes-Guridi I., França Santos M., Vedral V. (2004). Spin-1/2 Geometric Phase Driven by Decohering Quantum Fields. Phys. Rev. Lett..

[175] Bose S., Carollo A., Fuentes-Guridi I., França Santos M., Vedral V. (2003). Vacuum induced berry phase: Theory and experimental proposal. J. Mod. Opt..

[176] Alcalde M.A., Pimentel B. (2011). Path integral approach to the full Dicke model. Phys. A Stat. Mech. Its Appl..

[177] Boneberg M., Lesanovsky I., Carollo F. (2022). Quantum fluctuations and correlations in open quantum Dicke models. Phys. Rev. A.

