# Correction: Herrera Romero, R.; Bastarrachea-Magnani, M.A. Phase and Amplitude Modes in the Anisotropic Dicke Model with Matter Interactions. *Entropy* 2024, *26*, 574

**DOI:** 10.3390/e26110965

**Published:** 2024-11-11

**Authors:** Ricardo Herrera Romero, Miguel Angel Bastarrachea-Magnani

**Affiliations:** Departamento de Física, Universidad Autónoma Metropolitana-Iztapalapa, Av. Ferrocarril San Rafael Atlixco 186, Mexico City C.P. 09310, Mexico

The authors wish to make the following correction to this published paper [1]. There was a mistake over Equation (9): some factors were missing over the *x*-interactions from the Holstein-Primakoff expansion and were lost in the substitution. It should read

(9) $$\begin{array}{c} \hat{H}=\omega {\hat{c}}^{†}\hat{c}+\left[\omega_{0}+\gamma \frac{\alpha \beta }{\sqrt{k}}\left(1+\xi \right)+\eta_{z}\left(2\beta^{2}-1\right)-\beta^{2}\eta_{x}\right]{\hat{d}}^{†}\hat{d}\\ +\left[\omega \alpha -\gamma \sqrt{k}\beta \left(1+\xi \right)\right]\sqrt{2j}\left({\hat{c}}^{†}+\hat{c}\right)\\ +\left[-\omega_{0}\beta +\gamma \alpha \left(\frac{k-\beta^{2}}{\sqrt{k}}\right)\left(1+\xi \right)-\eta_{x}k\beta \left(1-\frac{\beta^{2}}{k}\right)-\eta_{z}\left(2\beta^{2}-1\right)\beta \right]\sqrt{2j}\left({\hat{d}}^{†}+\hat{d}\right)\\ +\left[\gamma \frac{\alpha \beta }{4\sqrt{k}k}\left(2-\beta^{2}\right)\left(1+\xi \right)+\eta_{x}\frac{k}{4}\left(1-\frac{4\beta^{2}}{k}\right)+\eta_{z}\beta^{2}\right]{\left({\hat{d}}^{†}+\hat{d}\right)}^{2}-\eta_{y}\frac{k}{4}{\left({\hat{d}}^{†}-\hat{d}\right)}^{2}\\ -\gamma \left[\frac{\beta^{2}}{2\sqrt{k}}\left(1+\xi \right)\right]\left({\hat{c}}^{†}+\hat{c}\right)\left({\hat{d}}^{†}+\hat{d}\right)+\gamma \sqrt{\xi_{0}}\left[\left(\hat{c}d^{†}+{\hat{c}}^{†}\hat{d}\right)+\xi \left({\hat{c}}^{†}{\hat{d}}^{†}+\hat{c}\hat{d}\right)\right]\\ +\left[\omega \alpha^{2}+\omega_{0}\beta^{2}-\frac{\omega_{0}}{2}-\gamma \sqrt{k}2\alpha \beta \left(1+\xi \right)+\eta_{x}k\beta^{2}+\eta_{z}{\left(\beta^{2}-\frac{1}{2}\right)}^{2}\right]2j\\ -\gamma \frac{\alpha \beta }{2\sqrt{k}}\left(1+\xi \right)+\eta_{x}\frac{\beta^{2}}{2}, \end{array}$$

As a result, the following expressions are modified, including reduced variables expressed in the third paragraph of Section 3.2 in the original paper:

$$\begin{array}{c} \omega_{A}=\frac{\omega_{0}}{2}\left(\frac{1}{\mu_{x}}-\frac{\eta_{z}}{\omega_{0}}\right)\left(1+\mu_{x}\right),\\ \omega_{B}=\frac{\omega_{0}}{2}\frac{1-\mu_{x}}{4}\left[\frac{1}{1+\mu_{x}}\frac{3+\mu_{x}}{\mu_{x}}+\frac{\eta_{z}}{\omega_{0}}\frac{1+3\mu_{x}}{1+\mu_{x}}+\frac{\eta_{x}}{\omega_{0}}\frac{4\mu_{x}^{2}}{1-\mu_{x}^{2}}\right],\\ \omega_{C}=-\frac{\omega_{0}}{8}\frac{\eta_{y}}{\omega_{0}}\left(1+\mu_{x}\right),\;\;\;\omega_{D}=-\frac{\sqrt{2}}{4}f_{\xi x}^{1/2}\sqrt{\omega \omega_{0}}{\tilde{\omega }}_{zx}\frac{1-\mu_{x}}{\sqrt{1+\mu_{x}}},\;\;\;\omega_{E}=\gamma \sqrt{\frac{1}{2}\left(1+\mu_{x}\right)},\\ \omega_{F}=-\frac{\omega_{0}}{2}\left\{\left[\frac{1-\mu_{x}^{2}}{2\mu_{x}}+\left(\mu_{x}-\frac{\eta_{z}}{2\omega_{0}}\right)\right]2j+\frac{1}{2}\left[\frac{1}{\mu_{x}}-\frac{\eta_{z}}{\omega_{0}}\right]\left(1-\mu_{x}\right)\right\}. \end{array}$$

Due to the modification in $\omega_{F}$, Equation (34) is rewritten as

(34) $$\begin{array}{c} \epsilon_{1}=\omega_{0}\left[\frac{1-\mu_{x}^{2}}{2\mu_{x}}+\left(\mu_{x}-\frac{\eta_{z}}{2\omega_{0}}\right)\right]2j+\omega_{0}\left(\frac{1}{\mu_{x}}-\frac{\eta_{z}}{\omega_{0}}\right)+\omega \end{array}$$

This impacts some of the results. First, after correcting the expressions, Figure 3(a1), (b1), and (c1) in the original paper are slightly modified without significant qualitative differences, as shown here in Figure 3.

The figure is a correction of Figure 3(a1–c1) in Ref. [1].

Second, the discussion about how the phase and amplitude modes behave toward the deformed phase in Section 3.3 in the original paper, must be modified. Equation (43) in the original paper changes to

(43) $$\begin{array}{c} \epsilon_{2\pm }^{N}=\sqrt{1-\left(1\mp 1\right)\frac{\Delta \eta_{zy}}{2\omega_{0}}},\;\;\;\;\epsilon_{2\pm }^{S}=\sqrt{\frac{\left(1\mp 1\right)\left(1-\frac{\Delta \eta_{zy}\mu_{x}}{\omega_{0}}\right)+\left(1\pm 1\right)\left(1-\frac{\eta_{z}\mu_{x}}{\omega_{0}}\right)}{2\left(1-\frac{\eta_{z}\mu_{x}}{\omega_{0}}\right)}}. \end{array}$$

We observe that the phase mode becomes undefined when $\Delta \eta_{zy}>\omega_{0}$, or $\Delta \eta_{zy}\mu_{x}>\omega_{0}$ in the normal and superradiant phases, respectively. For the amplitude mode we have $\epsilon_{2-}^{N,S}=1$, so its energy just becomes $\epsilon_{-}^{N,S}=\epsilon_{1-}^{N,S}$. The paragraph after Equation (43) should be read:

Because $\epsilon_{\pm }^{S,N}=\epsilon_{2\pm }^{S,N}\epsilon_{1\pm }^{S,N}$, the phase mode becomes undefined when $\Delta \eta_{zy}\geq \omega_{0}$, or $\Delta \eta_{zy}\mu_{x}\geq \omega_{0}$ in the normal and superradiant phases, respectively, and the amplitude mode turns to $\epsilon_{-}^{N,S}=\epsilon_{1-}^{N,S}$.

Finally, the amplitude mode at the critical coupling $\gamma_{\xi x}^{c}$ might become discontinuous. In the normal phase, Equation (44) in the article remains unchanged. We reproduce it here for comparison. However, in the superradiant phase, it becomes:

(44) $$\begin{array}{c} \epsilon_{+}^{N,c}=\sqrt{\frac{1}{2}\left(\omega^{2}+\omega_{0}^{2}\omega_{zx}^{2}\right)\left[\left(1+{\tilde{\omega }}_{zy}^{2}\right)+\sqrt{{\left(1-{\tilde{\omega }}_{zy}^{2}\right)}^{2}+4{\tilde{\omega }}_{zx}^{2}\frac{{\left(1-\xi \right)}^{2}}{{\left(1+\xi \right)}^{2}}}\right]}, \end{array}$$

in the normal phase, and by

(45) $$\begin{array}{c} \epsilon_{+}^{S,c}=\sqrt{\frac{1}{2}\left[\omega^{2}+\left(1-\frac{\eta_{z}}{\omega_{0}}\right)\omega_{0}^{2}{\tilde{\omega }}_{zx}^{2}\right]\left[\left(1+\frac{{\tilde{\omega }}_{zy}^{2}}{1-\frac{\eta_{z}}{\omega_{0}}}\right)+\sqrt{{\left(1-\frac{{\tilde{\omega }}_{zy}^{2}}{1-\frac{\eta_{z}}{\omega_{0}}}\right)}^{2}+\frac{4{\tilde{\omega }}_{zx}^{2}}{1-\frac{\eta_{z}}{\omega_{0}}}\frac{{\left(1-\xi \right)}^{2}}{{\left(1+\xi \right)}^{2}}}\right]} \end{array}$$

in the superradiant phase. Discontinuities may arise for $\eta_{z}\neq 0$ at the critical coupling. In the absence of interactions it becomes

(46) $$\begin{array}{c} \epsilon_{+}^{c}=\sqrt{\left(\omega^{2}+\omega_{0}^{2}\right)\left(1+\left|\frac{1-\xi }{1+\xi }\right|\right)}, \end{array}$$

Discontinuities may arise for $\eta_{z}\neq 0$ at the critical coupling, but for $\eta_{z}=0$ we recover the results in this article. With this correction, the order of some equations has been adjusted accordingly.

The paper suffered from minor quantitative calculation mistakes, also resulting in updating Figure 3 within it, but all qualitative argumentation and conclusions remain unchanged. This correction was approved by the Academic Editor. The original publication has also been updated.

## Figures and Tables

**Figure 3:**
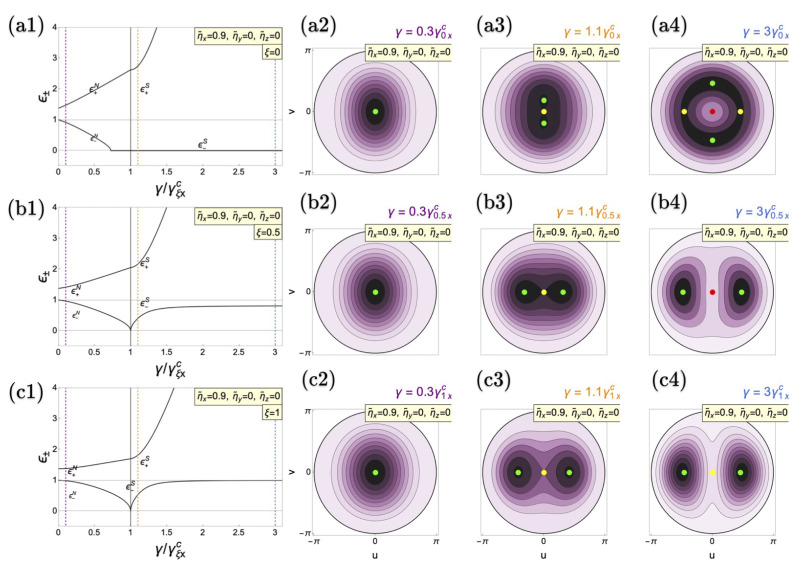
Polariton modes of the anisotropic Dicke model as a function of the coupling for (**a1**) TC limit (ξ=0), (**b1**) anisotropic case (ξ=0.5), and (**c1**) Dicke limit (ξ=1) with material collective interactions at $\eta_{x}=0.9\omega_{0}$ ($\Delta \eta_{zy}=0.0$, $\Delta \eta_{zx}=-0.9\omega_{0}$). The critical coupling $\gamma_{\xi x}^{c}$ ($\gamma_{\xi y}^{c}$) is indicated by the vertical solid black (dotted red) line. (**a2**–**a4**,**b2**–**b4**,**c2**–**c4**) depict the corresponding energy surfaces for the respective cases. The vertical dashed purple line shows the position of energy surfaces in the energy spectrum in the normal phases (**a2**–**c2**). The yellow one indicates the location of energy surfaces in the superradiant phase (**a3**–**c3**), while the blue line represents higher values of light–matter couplings (**a4**–**c4**). Green points in the energy surfaces represent energy minima, red ones indicate maxima and yellow points denote saddle points. Tilde variables are scaled to $\omega_{0}$. All cases are calculated in resonance ($\omega =\omega_{0}=1$).
